# Mitochondria-derived methylmalonic acid aggravates ischemia–reperfusion injury by activating reactive oxygen species-dependent ferroptosis

**DOI:** 10.1186/s12964-024-01479-z

**Published:** 2024-01-18

**Authors:** Junchen Guo, Shanjie Wang, Xin Wan, Xiaoxuan Liu, Zeng Wang, Chenchen Liang, Zhenming Zhang, Ye Wang, Miao Yan, Pengyan Wu, Shaohong Fang, Bo Yu

**Affiliations:** 1 Department of Cardiology, Second Affiliated Hospital of Harbin Medical University, Nangang District, Harbin, 150000 China; 2 The Key Laboratory of Myocardial Ischemia, Chinese Ministry of Education, Nangang District, Harbin, 150000 China; 3 Department of Cardiology and Shanghai Institute of Precision Medicine, Ninth People’s Hospital, Shanghai Jiao Tong University School of Medicine, Shanghai, 200000 China

**Keywords:** Ischemia reperfusion, Methylmalonic acid, Oxidative stress, Ferroptosis, KEAP1/NRF2

## Abstract

Ferroptosis is a regulatory cell death process pivotal in myocardial ischemia–reperfusion (I/R) injury. However, the precise mechanism underlying myocardial ferroptosis remains less known. In this study, we investigated the pathophysiological mechanisms of methylmalonic acid (MMA) associated with ferroptosis activation in cardiomyocytes after I/R. We found an increase level of MMA in patients with acute myocardial injury after reperfusion and AC16 cells under hypoxia/reoxygenation (H/R) condition. MMA treatment was found to be associated with excessive oxidative stress in cardiomyocytes, leading to ferroptosis-related myocardial injury. In mice with I/R injury, MMA treatment aggravated myocardial oxidative stress and ferroptosis, which amplified the myocardial infarct size and cardiac dysfunction. Mechanistically, MMA promoted NOX2/4 expression to increase reactive oxygen species (ROS) production in cardiomyocytes, aggravating myocardial injury. Notably, the increased ROS further activated ferroptosis by inhibiting solute carrier family 7 member 11 (SLC7A11) and glutathione peroxidase 4 (GPX4) expression. In addition, MMA decreased the ectopic nuclear distribution of nuclear factor E2-related factor 2 (NRF2) by increasing the interaction between NRF2 and kelch-like ECH-associated protein 1 (KEAP1). This impeded the activation of GPX4/SLC7A11, downstream of NRF2, activating ferroptosis and aggravating myocardial cell injury. Collectively, our study indicates that MMA activates oxidative stress and ROS generation, which induces ferroptosis to exacerbate cardiomyocyte injury in an I/R model. These findings may provide a new perspective for the clinical treatment of I/R injury and warrant further investigation.

**Supplementary Information:**

The online version contains supplementary material available at 10.1186/s12964-024-01479-z.

## Introduction

Acute myocardial infarction (AMI) poses a major risk to human health worldwide [1, 2]. Although percutaneous coronary intervention is necessary to recanalize occluded vessels [3, 4], it can provoke ischemia–reperfusion (I/R) injury in the heart [5]. I/R injury triggers various pathophysiological pathways, causing irreversible cardiomyocyte injury and expanding the size of myocardial infarction [6]. Therefore, exploring the molecular mechanisms underlying I/R injury is clinically important.

Reactive oxygen species (ROS), generated during cellular metabolism, are among the primary contributors to post-ischemic injury [7]. Previous research has confirmed a considerable reduction in myocardial cell injury in isolated rat hearts upon exposure to superoxide dismutase and catalase during myocardial I/R [8]. ROS activation also triggers lipid chain peroxidation, altering the structural and functional integrity of cells and resulting in cell necrosis and apoptosis [9–11].

Ferroptosis is a novel type of programmed cell death characterized by the accumulation of iron-dependent lipid peroxides [12]. The hallmarks of ferroptosis primarily comprise mitochondrial shrinkage and increased membrane density, with normal nuclear morphology [12, 13]. This process contributes to the occurrence and development of myriad human diseases, with an important regulatory role in tumors, myocardial I/R, liver injury, and other diseases [13–17]. Moreover, the aberrant expression of essential proteins, such as solute carrier family 7 member 11 (SLC7A11) and glutathione peroxidase 4 (GPX4), impairs the antioxidant capacity of cells, leading to ferroptosis [18, 19]. Additionally, nuclear factor E2-related factor 2 (NRF2), an upstream regulator of GPX4, is a vital transcription factor that regulates the antioxidant stress response [20]. Under normal conditions, NRF2 and kelch-like ECH-associated protein 1 (KEAP1) form a complex in the cytoplasm to generate polymers that dissociate under oxidative stress [21]. Dissociated NRF2 enters the nucleus and activates downstream antioxidant target genes [22]. Indeed, the overexpression of NRF2 and its downstream genes substantially reduces ROS levels in the myocardial cell cytoplasm and mitochondria [23]. Moreover, promoting GPX4 and NRF2 expression inhibits I/R-induced cardiac myocyte ferroptosis [23, 24].

Methylmalonic acid (MMA) is an intermediate metabolite derived from the mitochondria and generated via methylmalonic acid CoA degradation. Methylmalonyl-CoA is allosterically converted to succinyl-CoA via the catalytic activity of the mitochondrial methylmalonyl-CoA mutant enzyme and participates in the tricarboxylic acid cycle [25–28]. MMA interferes with mitochondrial energy production, leading to a redox imbalance by directly inhibiting electron transport complexes and α-ketoglutarate dehydrogenase [29, 30]. In addition, owing to its similar chemical structure, the effect of MMA resembles the mitochondrial toxic effect of malonate, a respiratory chain complex inhibitor [31]. Animal and clinical studies have reported that the methylmalonic acid hematic disease deleteriously affects mitochondrial morphology and function [31]. Furthermore, recent studies have confirmed MMA-induced mitochondrial morphological abnormalities and redox disorders in renal tubular cells [30]. MMA-triggered mitochondrial dysfunction primarily involves the liver, kidneys, and nervous system. However, although the heart contains abundant mitochondria, the significance of MMA in the cardiovascular system has not been fully elucidated, and its role in cardiac disorders remains unclear.

Our recent study showed the favorable predictive value of circulating MMA regarding the 10-year mortality risk of patients with cardiovascular disease beyond the established biomarkers—C-reactive protein and homocysteine [32]. However, the specific underlying mechanism has not been reported. Accordingly, in this study, we investigate the pathophysiological mechanisms of MMA regarding the activation of ferroptosis in cardiomyocytes after I/R.

## Methods

### Study participants

Fifty participants with AMI were recruited from Harbin Medical University Second Affiliated Hospital, Harbin Medical College, Harbin, China, between March 2017 and December 2018. Blood samples were collected before and after revascularization surgery. Fifteen healthy control subjects with no significant systemic diseases (such as cancer, pulmonary disease, or infectious diseases) were also recruited from Harbin Medical University Second Affiliated Hospital. The research protocol was approved by the Ethics Committee of HMUSAH (KY2017-249) and performed in accordance with the Declaration of Helsinki. Informed consent was obtained from all the participants. Following the collection of blood samples, plasma was immediately separated via centrifugation and stored at − 80 °C until analysis.

### Animals and experimental protocol

C57BL/6 J mice were obtained from the Experimental Animal Center of the Harbin Medical University. Adult male mice (8–12 weeks of age, 20–25 g) were housed in a facility with a 12 h light/12 h dark cycle at 23 ± 3 °C and 30–70% humidity. All experiments were performed in accordance with the Guiding Principles for the Care and Use of Animal Laboratories at Harbin Medical University and were approved by the Ethics Committee for Animal Experimentation of the School of Pharmacy, Harbin Medical University.

C57BL/6 J mice were randomized into different groups and treated as follows: Control (saline, ip.), MMA (400 mg/kg/d for 7 days, ip.), MMA + NAC [(300 mg/kg of NAC on day 2, 4, and 6 of the 7 days, ip.) + MMA (400 mg/kg/d for 7 days, ip.)], MMA + FER-1 [(10 mg/kg of FER-1 on day 2, 4, and 6 of the 7 days, ip.) + MMA (400 mg/kg/d for 7 days, ip.)] and MMA + RSL3 [(10 mg/kg of RSL3 on day 2, 4, and 6 of the 7 days, ip.) + MMA (400 mg/kg/d for 7 days, ip.)]. MMA (Methylmalonic acid; 516–05-2, MedChemExpress, USA), NAC (N-acetylcysteine; MedChemExpress, 616–91-1, USA), FER-1 (Ferrostatin-1; MedChemExpress, 347,174–05-4, USA) and RSL3 ((1S,3R)-RSL3; MedChemExpress, 1,219,810–16-8, USA) were dissolved in a mixture of saline, Tween 80 (HY-Y1891, MedChemExpress, USA) and PEG300 (HY-Y0873, MedChemExpress, USA), and the PH was adjusted to 7.4 by HCL or NAOH before intraperitoneal injection. All the mice then underwent sham or I/R surgery. The investigator was blinded to the group allocation and assessing the outcome during the experiments.

### I/R model and infarct size measurements

Mice were anesthetized with 2% avertin (0.1 mL/10 g of body weight). Cardiac I/R injury was induced by ligating the left anterior descending (LAD) coronary artery using a 7–0 silk suture for 45 min, followed by reperfusion for 24 h. Sham-group mice were subjected to the same surgical procedure without LAD ligation. Next, the LAD was retied, and then the heart was perfused with 1 mL of 1% Evans blue (Biofroxx, EZ5679B136, Germany) to visualize the area at risk (AAR). The heart was rapidly excised and serially sectioned to a thickness of 1–2 mm. The slices were incubated with 2.0% 2,3,5-triphenyl tetrazolium chloride (Solarbio, Cat#G3005, China) at 37 °C for 15 min to measure the infarct area. Staining was stopped using ice-cold sterile saline, and the slices were fixed in 4% neutral-buffered formaldehyde for 5 min. Each side of each slice was photographed digitally using a somatic microscope (ZEISS Discovery.V8). The infarct area (pale area) and AAR (pale area plus pink area) were measured using computerized planimetry (ImageJ v1.51). The infarct size was calculated as the infarct area divided by the AAR.

### Pathological staining

Heart tissue were fixed with 4% paraformaldehyde at room temperature for 72 h, paraffin-embedded, and sliced into sections with 4 μm thickness. Sections was stained with HE (Right tech, China) staining as previously described [33].

### MMA measurement

Peripheral vein blood samples were collected and the monocytes, platelets, and plasma were separated. Plasma samples were collected after centrifugation at 1500 g and 100 μL of the sample was used for quality control analysis. Another 100 μL of plasma was taken and added to 400 μL acetonitrile and then fully oscillated for 3 min and placed in an ice bath for 15 min. After high-speed centrifugation at 4 ℃ at 12,000 rpm for 15 min, 100 μL of supernatant was added to 100 μL of acetonitrile aqueous solution (75/25, v/v) for resolution in an equal volume. After oscillating for 1 min, 85 μL of the supernatant was transferred to the sample bottle after centrifugation at 12,000 rpm for 15 min at 4 ℃. An UHPLC-Q-Orbitrap (Thermo Fisher Scientific, USA) was used to determine the MMA levels in the samples.

For mice heart tissue, an amount of 0.05 g of heart tissue was mixed with 500 µL of 70% methanol/water. For mice serum, 50 μL of the sample was mixed with 250 μL of 20% acetonitrile/methanol. For AC16 cells, the sample was mixed with 500 µL of 80% methanol/water (precooled at -20 °C). All the samples were vortexed for 3 min and centrifuged at 12,000 r/min for 10 min at 4 °C. Take 280 μL of supernatant into a new centrifuge tube and place the supernatant in -20 °C refrigerator for 30 min. Then the supernatant was centrifuged again at 12,000 r/min for 10 min at 4 °C. After centrifugation, 180 μL of supernatan were analyzed using an LC–ESI–MS/MS system (UPLC, ExionLC AD, https://sciex.com.cn/; MS, QTRAP® 6500 + System, https://sciex.com/). The analytical conditions were as follows, HPLC: column, ACQUITY HSS T3 (i.d.2.1 × 100 mm, 1.8 μm); solvent system, water 0.05% formic acid (A), acetonitrile with 0.05% formic acid (B); The gradient was started at 5% B (0 min), increased to 95% B (8–9.5 min), finally ramped back to 5% B (9.6–12 min); flow rate, 0.35 mL/min; temperature, 40 °C; injection volume: 2 μL.

### Echocardiography

Two-dimensional echocardiography was performed on anesthetized mice (3.0% isoflurane with O_2_ flow at 1.0 L/min) using a Vevo 3100LT echo with an MS400 transducer. Echocardiographic images were recorded along the parasternal short axis and stored for offline analysis (Vevo 3100 software) by two blinded observers. The echocardiography software was used to calculate the left ventricular ejection fraction (EF), fractional shortening (FS), left ventricular end-systolic diameter (LVIDs), left ventricular end-diastolic diameter (LVIDd), left ventricular anterior wall; systolic (LVAWs) and left ventricular anterior wall; diastolic (LVAWd).

### Transmission electron microscopy (TEM)

Heart tissue were fixed with 2.5% glutaraldehyde, 2.5% polyvidone 25, and 0.1 M sodium cacodylate (pH 7.4). After washing with 0.1 M sodium cacodylate buffer (pH 7.4), the samples were post-fixed in the same buffer containing 2% osmium tetroxide and 1.5% potassium ferrocyanide for 1 h. The samples were rinsed once in water, stained en bloc with uranyl acetate, dehydrated using an ascending ethanol series, and embedded in Durcupan ACM-based resin. Ultrathin sections were cut using a Reichert Ultracut S ultramicrotome (Science Service, Munich, Germany), and lead citrate was used for contrast. Images were captured using an EM 10 CR electron microscope (H-7650; Hitachi, Tokyo, Japan) and analyzed by an independent blinded investigator.

### Cell culture

Human AC16 cardiomyocyte cells were purchased from the China Center for Type Culture Collection. The cell lines used in this study were authenticated via short tandem repeat analysis and were regularly tested for mycoplasma. AC16 cells were cultured in Dulbecco’s modified Eagle’s medium (SH30022.01B, HYCLONE, USA) supplemented with 10% fetal bovine serum (0500, ScienCell, USA). PCR analysis was performed to obtain mycoplasma-free cells, which were cultured at 37 °C in a humid atmosphere containing 5% CO_2_.

### Cell viability assay

Cell viability was determined using the MTT Cell Proliferation and Cytotoxicity Assay Kit (C0009S, Beyotime Biotechnology, China). AC16 cells were seeded at a density of 5 × 10^3^ cells/well in 96-well plates and incubated overnight. AC16 cells were then treated as follows: MMA (12.5, 15, 17.5 and 20 mM for 3 h), DM-MMA (Dimethyl methylmalonate) (Aladdin, 609–02-9, China) (0.02, 0.05 and 0.1 and 0.2 μM for 3 h), DE-MMA (Diethyl Methylmalonate) (Aladdin, 609–08-5, China) (0.005, 0.01, 0.02 and 0.03 μM for 3 h), H_2_O_2_ (772,284–1, Aladdin, China) (200, 400, 600, 800 and 1000 μM for 6 h), MMA + NAC (5 μM NAC for 2 h followed by 3 h 17.5 mM MMA), MMA + FER-1 (2 μM FER-1 for 2 h followed by 3 h 17.5 mM MMA) or MMA + RSL3 (3 μM RSL3 for 2 h followed by 3 h 17.5 mM MMA). MMA, NAC, FER-1 and RSL3 were dissolved and diluted with PBS. Subsequently, 15 μL of MTT reagents (5 mg/mL) was added. Live cells were counted according to the optical density (OD) of each well and quantified using assay a microplate reader at 490 nm. The resulting OD was indicated as the percentage of cell viability in the control group, which was set at 100%.

### Hypoxia/reoxygenation (H/R) model

H/R was induced to simulate in vivo I/R injury in myocardial cells. AC16 cells were exposed to hypoxia for 6 h in a hypoxic incubator (5% CO_2_ and 1% O_2_ at 37 °C), in which O_2_ was replaced with N2. After hypoxia exposure, the cells were subjected to reoxygenation for 24 h at 37 °C in a normoxic incubator with 95% air and 5% CO_2_.

### ROS detection

The ROS generation in cells after MMA treatment was evaluated based on the fluorescence intensity of dihydroethidium (DHE), 5-(and-6)-chloromethyl-2′,7′-dichlorodihydrofluorescein diacetate (DCFH-DA), and a MitoSOX probe. The cells were harvested after MMA treatment, washed with PBS, and incubated with 5 μM DHE (S0063, Beyotime, China), 5 mg/mL DCFH-DA (S0033S, Beyotime, China), or 1 μM of the MitoSOX (MAN0028459, ThermoFisher, USA) probe for 30 min at 37 °C in the dark. For mice, the frozen sections were pretreated with PBS for 10 min and then incubated with DHE staining solution (10 µmol/L) at 37 °C in the dark cassette for 30 min. Then, heart sections were washed with PBS three times and remained wet until observation. The fluorescent signals were measured using a confocal laser scanning microscope (ZEISS LSM 700) and a FACSVerse flow cytometer (BD, USA).

### Lipid peroxidation staining

Lipid peroxidation in the cells after MMA treatment was evaluated based on the fluorescence intensity of the Liperfluo probe. In brief, the cells were harvested after MMA treatment, washed with PBS, and incubated with 5 μM of the Liperfluo (L248, Dojindo, China) probe for 30 min at 37 °C in the dark. After rinsing, fluorescent signals were measured using a confocal laser scanning microscope (ZEISS LSM 700).

### JC-1 staining

Mitochondrial membrane potential was measured using JC-1 (C2003S, Beyotime, China) fluorescence imaging. The AC16 cells were incubated with JC-1 solution for 20 min at 37 °C and then rinsed twice with the JC-1 buffer. Images were obtained using a confocal laser scanning microscope (ZEISS LSM 700). The ratio of JC-1 aggregates to JC-1 monomer fluorescence represented the mitochondrial membrane potential.

### Mitochondrial imaging

The mitochondria were stained with MitoTracker Red (C1035, Beyotime, China). The AC16 cells were incubated with Mito-Tracker Red (200 nM) solution for 30 min at 37 °C and washed three times with PBS; images were obtained using a confocal laser scanning microscope (ZEISS LSM 700).

### Immunofluorescence and TdT-mediated dUTP nick end labeling (TUNEL)

Immunofluorescence frozen slides were air dried at room temperature (RT) for 10 min and fixed with cold acetone (− 20 °C) for 10 min. After rinsing three times in distilled water, slides were first incubated with 0.3% Triton-X 100 for 30 min at 37 °C and then with primary antibody at 4 °C overnight. On day 2, the slides were rewarmed at RT for 10 min, followed by incubation with a secondary antibody at RT for 1 h. Finally, the nuclei were stained with 0.5 g/L DAPI for 10 min, and images were captured using a confocal laser scanning microscope (ZEISS LSM 700).

For the TUNEL assay (cat. No. E-CK-A320 and No. E-CK-A325, Elabscience, China), tissue slides were catalyzed with terminal deoxynucleotidyl transferase (TdT) enzyme and fluorescently labeled 2′-deoxyuridine 5′-triphosphate (dUTP) at 37 °C for 1 h. Nuclei were stained with DAPI. The number of TUNEL-positive cells was determined via fluorescence microscopy to determine the level of apoptosis.

### Transfection

Confluent cells were seeded 24 h before transfection. Cells were transfected with KEAP1 or methylmalonic CoA mutase (MMUT) small interfering RNAs (siRNA-KEAP1 or siRNA-MMUT) using Lip­3000 (L3000008, Invitrogen, USA) according to the manufacturer’s instructions. The transfected cells were incubated at 37 °C for 48 h, and the cellular proteins were extracted. The siRNAs used are listed in Supplementary Table 2. Western blot analysis was performed to confirm the efficiency of transfection (outlined in a subsequent section).

### Enzyme-linked immunosorbent assay (ELISA) and activity assay

Blood samples (0.8 mL) were extracted at the endpoints of experiments and maintained at RT for 30 min, followed by centrifugation at 3000 rpm for 15 min at 4 °C to facilitate supernatant collection. After treatment, the AC16 cells were collected via centrifugation. The quantification of MDA (malonaldehyde; E-EL-0060c, Elabscience, China), CK-MB (creatine kinase isoenzyme MB; E-E-M0355c, Elabscience, China), GSH (glutathione; E-BC-K030-M, Elabscience, China), ATP (E-BC-K157-M, Elabscience, China), and LDH (lactate dehydrogenase; E-BC-K046-M, Elabscience, China) in mouse serum or AC16 cells of different groups was performed using an ELISA kit or activity assay kit following the manufacturers’ protocols. MitoTEMPO (Aladdin, 1,334,850–99-5, China) was used to verify whether the mitochondrial targeted ROS inhibitor causes damage to cardiomyocytes.

### Co-immunoprecipitation

Protein lysates from cardiomyocytes (1000 μg of total protein) were incubated with 10 μg of anti-NRF2 monoclonal antibody overnight at 4 °C. Immune complexes were bound to protein A/G magnetic beads and collected using a magnetic stand. Proteins co-immunoprecipitated with NRF2 were eluted by heating at 95℃ for 5 min and then subjected to gel electrophoresis and immunoblotting.

### Western blot analysis

After treatment, cells were lysed in RIPA buffer containing protease and phosphatase inhibitors on ice. Nuclear and cytosolic fractions were separated using a nuclear/cytosol fractionation kit (R0050, Solarbio, China). The protein concentration was determined using a bicinchoninic acid (Beyotime, P0012, China) protein assay. Protein samples (30 μg) were separated via 10% or 12.5% sodium dodecyl sulfate–polyacrylamide gel electrophoresis and transferred to 0.22 μm PVDF membranes (FFP70, Beyotime, China), and blocked for 2 h at RT with 5% dried skimmed milk in Tris-buffered saline and 0.05% Tween 20. The membranes were then incubated with primary antibodies at 4 °C overnight. Subsequently, the membranes were incubated with horseradish peroxidase (HRP)-conjugated secondary antibodies (1:8000) or Mouse Anti-Rabbit IgG (conformation-specific) mAb HRP-conjugated secondary antibodies (1:2000, L27A9, CST, USA) for 1 h at RT. An ECL kit (MA0186-1-Jul-14H, Meilunbio, China) was used to visualize immunoreactivity using the ChemiDocTM MP Imaging System (Tanon, China). Protein bands were quantified using a Bio-Rad Chemi EQ densitometer and Bio-Rad Quantity One software (Tanon, China) and normalized to GAPDH or PCNA expression. The antibody dilution buffer was TBST (Tris-buffered saline with 0.1% Tween 20) containing 5% skim milk powder. The primary antibodies used in this study are listed in Supplementary Table 3.

### Real-time quantitative PCR (RT-qPCR)

Total RNA was extracted using TRIzol reagent (Thermo Fisher Scientific, USA) and reverse-transcribed with the RT Easy II First Strand cDNA Synthesis Kit (04379012001, Roche, Switzerland). cDNA (18 ng) was amplified via Real-Time PCR Easy (SYBR Green I) (HY-K0501, MCE, China) on an ED Sequence Detection system (ED, USA). The primers used are listed in Supplementary Table 4, and the gene expression values were normalized against those of *β-actin*.

### Statistical analysis

Statistical analyses were performed using statistical software R V.3.6.3 (R Foundation for Statistical Computing, Vienna, Austria) or GraphPad Prism 8.0. Continuous variables were expressed as the mean ± SD for normal distributions and as the median (25th, 75th percentile) for non-normally distributed data. Categorical variables were presented as numbers (percentages). Comparisons between groups were performed using Student’s *t*-tests, Mann–Whitney U tests, or Welch’s t-test for quantitative variables and the chi-squared test for categorical variables. A two-sided *p*-value < 0.05 was considered significant.

## Results

### MMA accumulation under ischemia–reperfusion

**Fig. 1:**
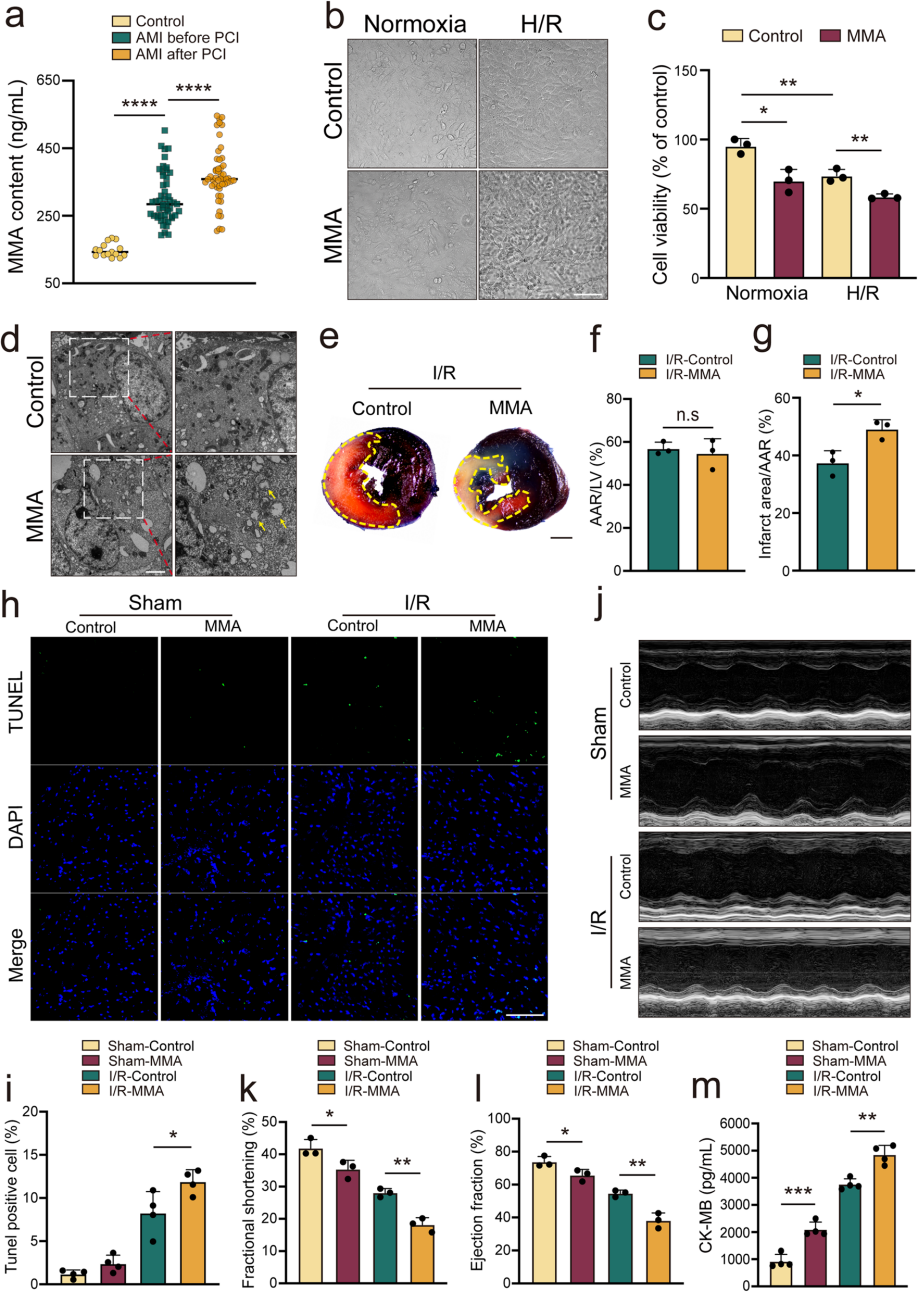
MMA accumulation aggravates myocardial injury. In vitro, AC16 cells were treated with MMA (17.5 mM) for 3 h and employed for further analyses. In vivo, mice were assigned to the sham operation or myocardial ischemia–reperfusion (I/R) injury group. MMA (400 mg/kg/d) was administered seven days before sham and I/R surgery. **a** MMA level of patients was detected by ultra-performance liquid chromatography-quadrupole-electrostatic field orbitrap (UHPLC-Q-Orbitrap) (*n* = 15 in control group; *n* = 50 in AMI before PCI/AMI after PCI group). **b** Morphology was evaluated by light microscopy after AC16 cells were treated with MMA (Scale bar: 50 μm). **c** AC16 cells were treated with/without MMA in Normoxia or hypoxia-reoxygenation (H/R) condition. Cell viability was detected by 3-(4,5-dimthyl-2-thiazolyl)-2,5-diphenyl-2-H-tetrazoliumbromide (MTT) assay (*n* = 3–6/group). **d** Representative TEM images of AC16 cells treated with/without MMA. Yellow arrows indicate mitochondria (Scale bar: 20 μm). **e** Representative images of heart sections with TTC/Evans staining (Scale bar: 2 mm). The area of AAR was marked with yellow dotted line. **f**, **g** Ratios of area at risk (AAR) to left ventricular (LV) area and infarct area to AAR are shown (*n* = 3/group). **h**, **i** Representative photomicrographs and averaged data of TdT-mediated dUTP nick end labeling (TUNEL) positive cells (*n* = 4/group) (Scale bar: 100 μm). **j** Cardiac damage was indicated as CK-MB assay (*n* = 4/group). **k** Representative images of M-mode echocardiograms. **l**, **m** Quantitative analysis of ejection fraction (EF) and fractional shortening (FS) echocardiography (*n* = 3/group). Data are expressed as the mean ± standard deviation. *P < 0.05, **P < 0.01, ***P < 0.001, ****P < 0.0001 and *n.s*, not significant

Targeted mass spectrometry was performed to determine plasma MMA levels in patients with/without AMI (Fig. 1a). The baseline characteristics of the study participants are shown in Supplementary Table 1. The finding revealed considerably enhanced MMA levels in the group of AMI before PCI and AMI after PCI compared with healthy controls. This finding suggested that in the presence of myocardial ischemia–reperfusion (I/R), MMA levels are elevated.

### MMA induces myocardial injury in vitro and in vivo

We used hypoxic reoxygenation (H/R) to simulate myocardial I/R and found that the MMA level of AC16 cells increased significantly after 6 h of hypoxia and 24 h of reoxygenation (Supplementary Fig. 1a). To assess the effect of elevated MMA levels on cardiomyocytes, we treated AC16 cells with various MMA concentrations. A reduction in cell viability was observed after AC16 cells were administered with MMA at concentrations ≥ 17.5 mM for 3 h (Supplementary Fig. 1b). A similar trend was observed when cardiomyocytes were treated with relatively low concentration of fat-soluble MMA [0.1 uM DM-MMA (Dimethyl methylmalonate) and 0.02 uM DE-MMA (Diethyl Methylmalonate)] (Supplementary Fig. 1c, d). In subsequent experiments, MMA was used at a concentration of 17.5 mM. The morphology of cardiomyocytes, as observed via light microscopy, was not significantly altered by H/R or MMA activation alone. However, simultaneous treatment with H/R and MMA caused the slight deformation of cardiomyocytes (Fig. 1b). However, MTT assays revealed that MMA affected cell activity under normoxic and H/R conditions (Fig. 1c). TEM confirmed the presence of mitochondrial swelling and disorganized cristae in MMA-treated AC16 cells, indicating that MMA might have adverse effects on cardiomyocytes (Fig. 1d).

To investigate further whether elevated levels of MMA cause damage to myocardial cells, we utilized siRNA to knockdown *MMUT* to induce MMA accumulation [34]. Western blotting revealed that MMUT-siRNA-2 effectively knocked down MMUT expression (Supplementary Fig. 1e, f). Therefore, MMUT-siRNA-2 and the control siRNA were used in subsequent experiments. Moreover, the elevated levels of CK-MB and LDH indicated that increased MMA damaged AC16 cells under normoxic and H/R conditions (Supplementary Fig. 1g, h). As expected, DM-MMA and DE-MMA have similar effects on cardiomyocytes (Supplementary Fig. 1i).

We further developed a mouse model of cardiac I/R injury to investigate the involvement of MMA in vivo. Consistently, MMA levels in the serum and myocardial tissue increased in mice in the I/R model compared to the Sham group (Supplementary Fig. 1j, k). Moreover, MMA treatment significantly amplified the I/R-induced infarct size in hearts subjected to 45 min of ischemia followed by 24 h of reperfusion (Fig. 1e–g). HE staining also showed that muscle fiber swelling and breakage was more obvious in I/R model mice with MMA intervention compared to the I/R group (Supplementary Fig. 1l). Meanwhile, DHE showed that MMA intervention further activated I/R-induced oxidative stress in mice heart (Supplementary Fig. 1m, n). Furthermore, an increased number of TUNEL-positive cells was observed in the myocardial tissue of mice in the IR-MMA group, as compared to the I/R-Control group. (Fig. 1h, i). Consistently, the worsening of cardiac dysfunction caused by MMA was evidenced by a decrease in the EF and FS (Fig. 1j–l) and elevated CK-MB in both the sham and I/R context (Fig. 1m). These results indicate that MMA might aggravate myocardial injury.

### MMA induces oxidative stress in cardiomyocytes

To further investigate the effect of MMA on mitochondrial, JC-1 staining revealed that MMA significantly reduced the mitochondrial membrane potential (Supplementary Fig. 2a–b). The ATP assay also showed that MMA reduced energy production (Supplementary Fig. 2c). In addition, RT-qPCR analysis revealed that MMA activated mitochondrial fission in AC16 cells by upregulating *DRP1*, *FIS1*, and *MFF* expression without significantly affecting mitochondrial fusion (Supplementary Fig. 2d). These results were supported by Western blotting, showing that MMA upregulated the protein levels of DRP1 while having minor effects on MFN1 in vitro (Supplementary Fig. 2e, f). Furthermore, MitoTracker Red staining showed that the mitochondrial network was disrupted in AC16 cells treated with MMA, as evidenced by a decreased mitochondrial length (Supplementary Fig. 2g). Consistently, MMA significantly impacted the mitochondrial structure in the I/R context and activated the mitochondrial division protein DRP1 in vivo (Supplementary Fig. 2h-j).

**Fig. 2:**
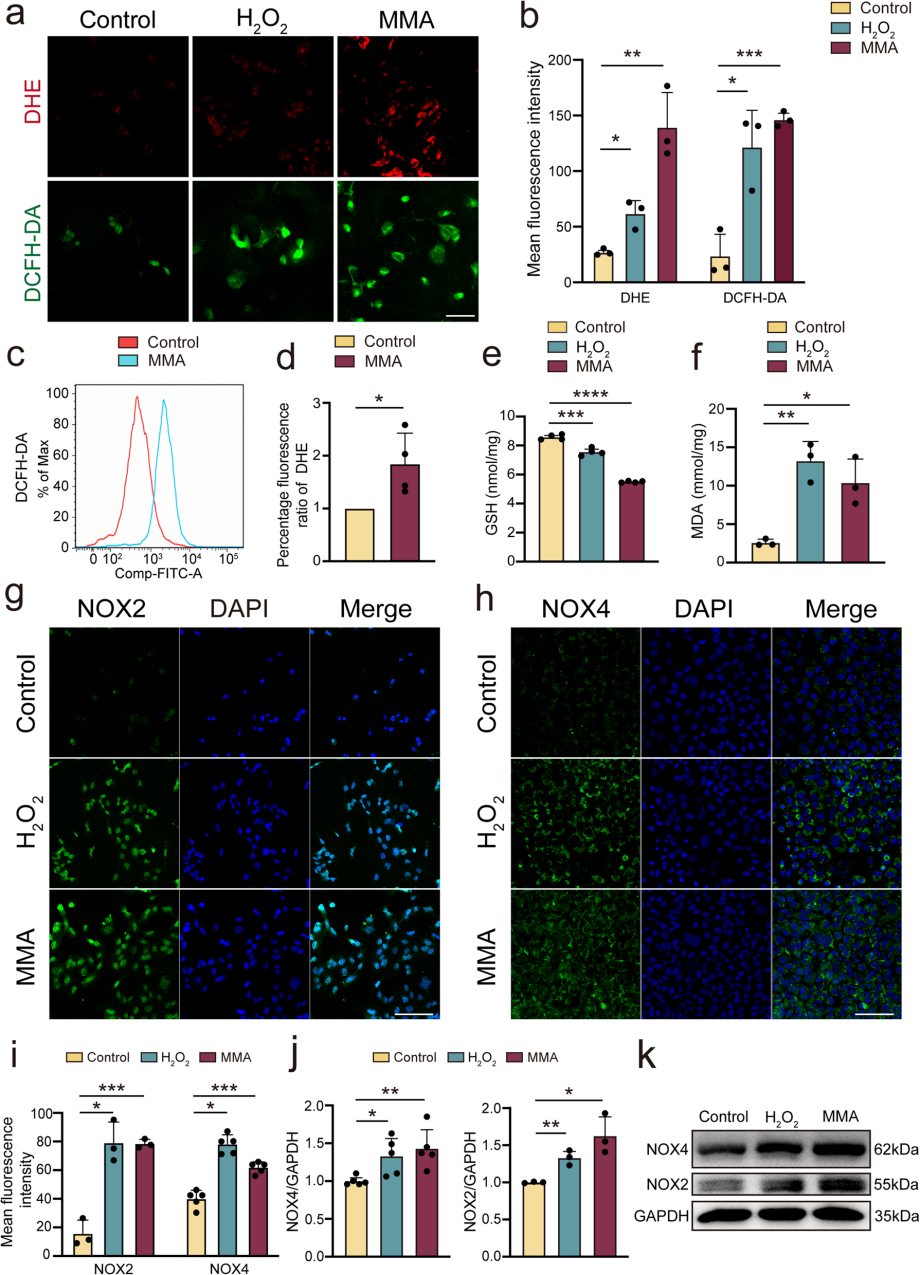
MMA-mediated ROS contributes to cardiomyocytes injury. In vitro, AC16 cells were treated with MMA (17.5 mM) for 3 h or H_2_O_2_ (600 μM) for 6 h and then used for further analyses. **a**, **b** ROS production was observed by DHE and DCFH-DA staining (*n* = 3/group) (Scale bar: 50 μm). **c** Flow cytometry was used to detect DCFH-DA fluorescence intensity under MMA treatment. **d** MMA increased the percentage of DHE-positive cells detected by flow cytometry (*n* = 4/group). **e**, **f** GSH and MDA were measured by ELISA kit in AC16 cells under MMA or H_2_O_2_ treatment (*n* = 3–4/group). **g**-**i** NOX2 and NOX4 expression in AC16 cells were analyzed by immunofluorescence staining (*n* = 3–5/group) (Scale bar: 100 μm). **j**, **k** Proteins were isolated from AC16 cells, and the levels of NOX2 and NOX4 were analyzed through western blot (*n* = 3–5/group). Data are expressed as the mean ± standard deviation. *P < 0.05, **P < 0.01, ***P < 0.001, ****P < 0.0001 and *n.s*, not significant

To investigate whether MMA affects mitochondrial and cardiac function by activating oxidative stress, we used H_2_O_2_ treatment of AC16 cells as a positive control. In subsequent experiments, H_2_O_2_ was utilized at a concentration of 600 μM, as administered with H_2_O_2_ at concentrations ≥ 600 μM for 6 h resulted in reduced cell viability (Supplementary Fig. 3a). Meanwhile, co-treatment with lower concentrations of H_2_O_2_ and MMA reduced cell viability, suggesting a potential synergistic effect between MMA and H_2_O_2_ in activating oxidative stress (Supplementary Fig. 3b). The intracellular ROS level in the H_2_O_2_ and MMA group was much higher than that in the control group, reflected by the increased DHE and DCFH-DA levels (Fig. 2a, b). Furthermore, immunofluorescence and flow cytometry results revealed higher levels of MitoSox in the MMA treatment group than control group, indicating that MMA triggered mitochondrial ROS production (Supplementary Fig. 3c–e). To determine whether MMA alone induced oxidative stress at the cellular level, flow cytometry was performed to detect the fluorescence intensity of DCFH-DA. MMA significantly enhanced this fluorescence signal in AC16 cells compared with the control group (Fig. 2c). Similarly, the percentage of DHE-positive cells was significantly increased 3 h after MMA treatment in AC16 cells (Fig. 2d).

GSH is an antioxidant tripeptide, containing a γ-amide bond and a sulfhydryl group. Both H_2_O_2_ and MMA reduced GSH levels, with MMA exhibiting a more prominent effect (Fig. 2e). MDA, comprising a series of complex compounds generated via oxidation–reduction reactions, can indirectly reflect lipid peroxidation. Either H_2_O_2_ or MMA significantly increased MDA levels in AC16 cells (Fig. 2f). The non-phagocytic cell oxidase (NOX) family constitutes NOX2 and NOX4, with NOX2 mainly producing ROS outside the mitochondria. In contrast, NOX4 generates ROS inside mitochondria. Immunofluorescence was used to detect NOX2 and NOX4 expression in vitro. The fluorescence intensities of NOX2 and NOX4 were significantly upregulated following MMA treatment, resembling the effect of H_2_O_2_, further indicating the activating effect of MMA on oxidative stress (Fig. 2g–i). In addition, western blotting results revealed that MMA significantly enhanced NOX2 and NOX4 expression in AC16 cells (Fig. 2j, k). These results suggest that MMA aggravates cardiomyocyte injury by activating pro-oxidative signals.

### The antioxidant NAC alleviates MMA-induced injury in cardiomyocytes

**Fig. 3:**
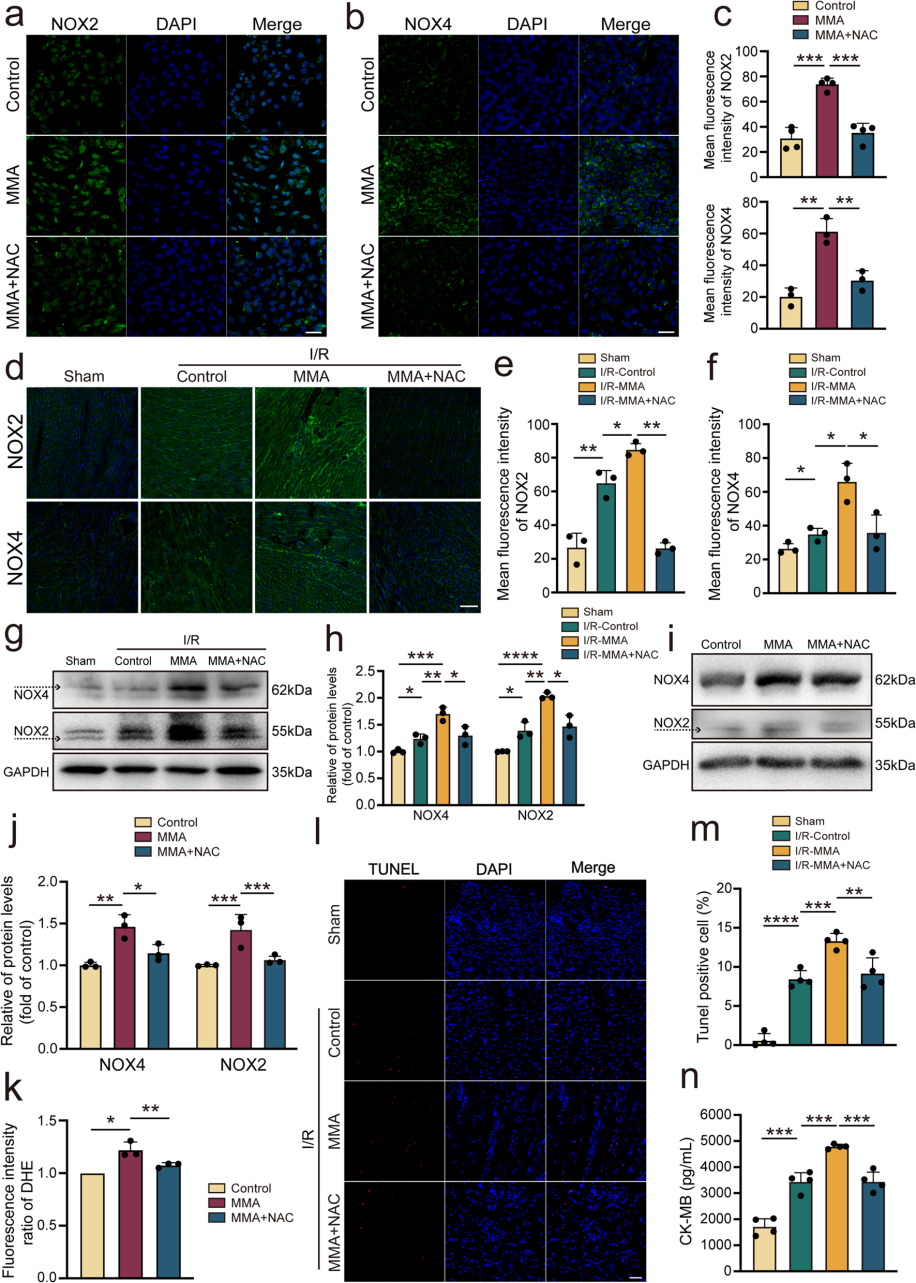
Inhibition of oxidative stress ameliorates MMA-mediated cardiomyocytes injury. In vitro, AC16 cells were treated with MMA (17.5 mM) alone for 3 h or with Acetylcysteine (NAC) (5 mM) for 2 h followed by MMA (17.5 mM) for 3 h. In vivo, mice were assigned to the sham operation or myocardial I/R injury group. MMA (400 mg/kg/d) was administered seven days before the I/R injury. Meanwhile, NAC (300 mg/kg) was administered three times before the I/R model (on day 2, 4, and 6 of the administration process). **a**-**c** NAC (5 mM) inhibited the expression of NOX2 and NOX4 in AC16 cells observed by confocal microscopy (*n* = 3–4/group) (Scale bar: 50 μm). **d**-**f** Immunofluorescence staining analysis to assess the NOX2 and NOX4 expression (*n* = 3/group) (Scale bar: 100 μm). **g**, **h** Western blotting analysis to determine NOX4 and NOX2 protein expression in the heart (*n* = 3/group). **i**, **j** Western blot assessed the NOX4 and NOX2 protein level in AC16 cells (*n* = 3/group). **k** NAC inhibited fluorescence intensity of DHE shown by Flow cytometry (*n* = 3/group). **l**, **m** Representative photomicrographs and averaged data of TUNEL positive cells (*n* = 4/group) (Scale bar: 50 μm). **n** NAC relieved myocardial damage assessed by CK-MB assay (*n* = 4/group). Data are expressed as the mean ± standard deviation. *P < 0.05, **P < 0.01, ***P < 0.001, ****P < 0.0001 and *n.s*, not significant

We further investigated whether MMA causes cardiomyocyte damage through oxidative stress. NAC, a cysteine precursor, is a common ROS inhibitor. The MTT assay demonstrated that NAC (5 mM) did not reduce cell viability (Supplementary Fig. 4a), nor did NAC (300 mg/kg) affect LDH activity or cardiac function (Supplementary Fig. 4b–i). To evaluate whether that NAC mediates the ROS-activated effects of MMA, mice were intraperitoneally injected with this MMA and NAC. As shown in Fig. 3a–c, NOX2 and NOX4 expression decreased after NAC treatment compared to the MMA group, where the mean fluorescence intensity was decreased. In line with the vitro data, MMA further upregulated the expression of NOX2 and NOX4 in the I/R model. In contrast, NAC reversed the MMA-induced overexpression of NOX2 and NOX4 (Fig. 3d–f). This phenomenon was further supported by changes in NOX2 and NOX4 protein levels in vivo and in vitro, as measured via western blotting, showing that NAC inhibited the expression of NOX2 and NOX4 in MMA-treated cells and mice upon I/R injury (Fig. 3g–j). Data from DHE staining further suggested that NAC decreased oxidative stress levels in AC16 cells (Fig. 3k).

NAC administration markedly decreased MMA-induced cardiomyocyte apoptosis (Fig. 3l, m). To further verify the relationship between oxidative stress and MMA-induced myocardial injury, we evaluated the degree of myocardial injury in mice by using a CK-MB ELISA kit. MMA further activated oxidative stress and aggravated cardiomyocyte injury based on the I/R model, as evidenced by the increased CK-MB levels; however, NAC intervention reversed these effects (Fig. 3n). Collectively, these data suggest that the antioxidant NAC might inhibit MMA-induced myocardial injury.

### MMA triggers ferroptosis through oxidative stress

**Fig. 4:**
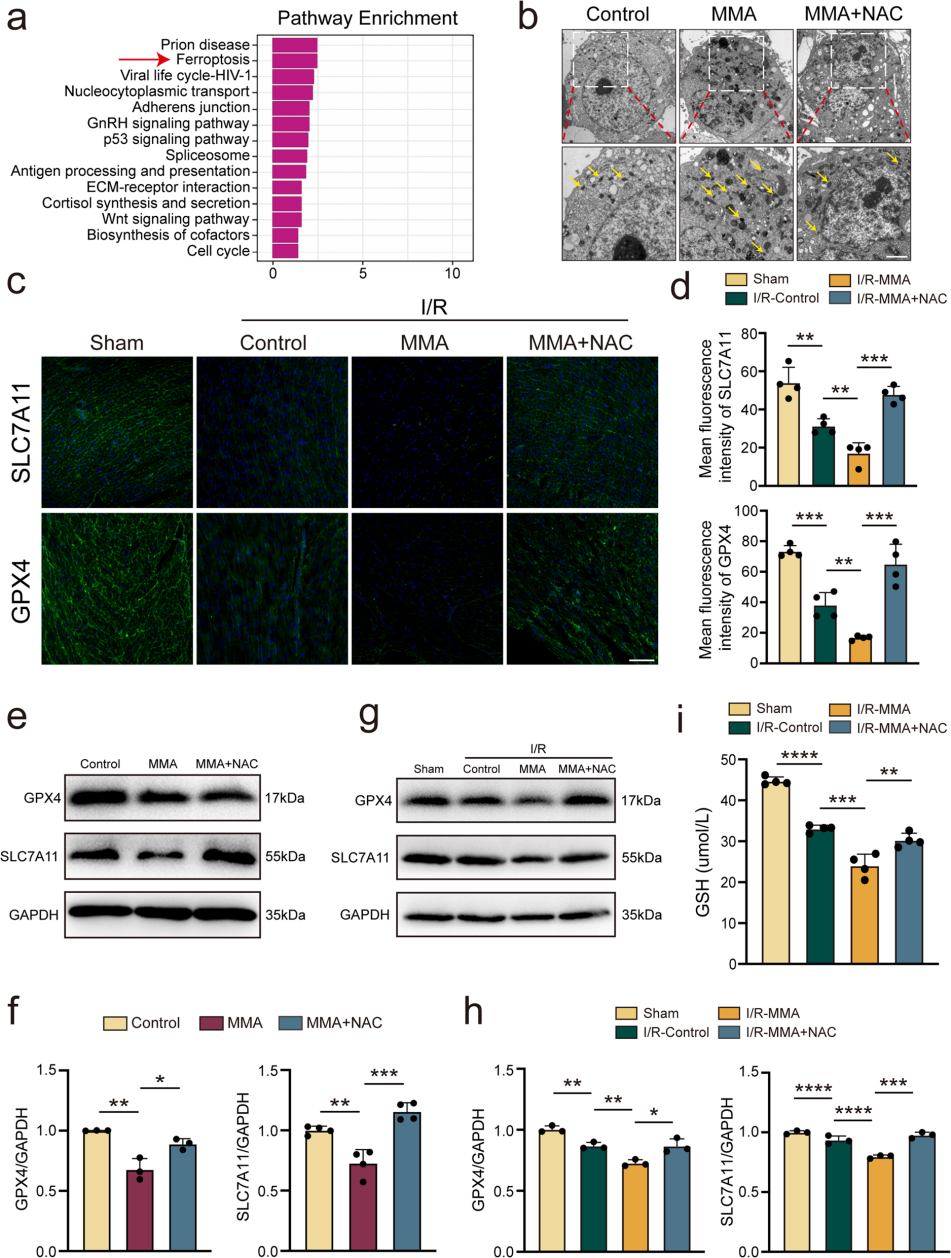
MMA triggers ferroptosis through oxidative stress. In vitro, AC16 cells were treated with MMA (17.5 mM) alone for 3 h or with NAC (5 mM) for 2 h followed by MMA (17.5 mM) for 3 h. In vivo, mice were assigned to the sham operation or myocardial I/R injury group. MMA (400 mg/kg/d) was administered seven days before the I/R injury. Meanwhile, NAC (300 mg/kg) was administered three times before the I/R model (on day 2, 4, and 6 of the administration process). **a** Pathway Enrichment showed a ferroptosis pathway associated with MMA treatment. **b** TEM analysis of mitochondria in AC16 cells. Yellow arrows indicate mitochondria (Scale bar: 20 μm). **c**, **d** Representative photomicrographs and averaged data of GPX4 and SLC7A11 expression in mice heart tissue (*n* = 4/group) (Scale bar: 100 μm). **e**, **f** Western blot analyzed the protein level of GPX4 and SLC7A11 in AC16 cells (*n* = 3–4/group). **g**, **h** Western blot analyzed the protein level of GPX4 and SLC7A11 in mice heart tissue (*n* = 3/group). **i** GSH was relieved by NAC in mice heart tissue. Data are expressed as the mean ± standard deviation. *P < 0.05, **P < *0.01*, ***P < 0.001, ****P < 0.0001 and *n.s*, not significant

Next, we investigated the potential mechanism by which MMA-induced oxidative stress aggravates cardiomyocyte injury. MMA treatment of AC16 cells enhanced the expression of *ACSL1*, *ACSL4*, *LPCAT3*, *PTGS2*, *NOCA4*, *IL33*, *BAK1*, *BID*, and *BAX* while decreasing that of *RIPK1* expression. Despite having no notable effect on *GSDMD* and *MCL1* expression, these findings suggest that MMA treatment could potentially trigger programmed cell death through ferroptosis, apoptosis, and necroptosis (Supplementary Fig. 5) [35–39]. Proteomic analysis revealed that ferroptosis was the most highly enriched pathway among the top 10 pathways associated with myocardial injury following MMA treatment (Fig. 4a). Furthermore, MMA induced lipid peroxidation, as evidenced by the increased intensity of Liperfluo, a fluorescent dye used for the specific detection of lipid peroxides (Supplementary Fig. 6a, b). The mitochondrial morphology of MMA treatment AC16 cells was also observed via TEM to assess whether the resulting oxidative stress promotes ferroptosis. Consistent with the results of previous study, TEM analysis revealed that smaller mitochondria might be a characteristic of ferroptosis, with increased membrane density [40]. AC16 cells in the MMA group exhibited more mitochondrial morphological abnormalities, indicating more pronounced ferroptosis (yellow arrows), which was inhibited by NAC treatment (Fig. 4b). Furthermore, as a targeted mitochondrial antioxidant, MitoTEMPO inhibited MMA-induced lipid peroxidation and improved MMA-induced myocardial cell damage, as evidenced by the decreased intensity of Liperfluo and the decreased levels of CK-MB and LDH under normoxic and H/R conditions (Supplementary Fig. 6c–f).

GPX4, the fourth member of the selenium-containing GPX family, is a promising indicator of ferroptosis, as is SLC7A11. Immunofluorescence staining revealed that I/R significantly inhibited the expression of SLC7A11 and GPX4 compared to the sham group. In addition, MMA treatment caused ferroptosis, as evidenced by the downregulation of SLC7A11 and GPX4 expression in the I/R model, whereas a considerable increase in SLC7A11 and GPX4 fluorescence intensity was detected in the I/R-MMA + NAC group (Fig. 4c, d). Similarly, NAC increased the expression of GPX4 and SLC7A11 in AC16 cells following MMA treatment (Fig. 4e, f). Western blot analysis revealed that the intraperitoneal injection of MMA inhibited the expression of GPX4 and SLC7A11 in myocardial tissue in the I/R model; however, this effect was reversed by NAC (Fig. 4g, h). Furthermore, MMA reduced GSH levels in the I/R model, which was reversed by NAC (Fig. 4i). These data indicate that MMA activates ferroptosis by upregulating oxidative stress.

### MMA inhibits NRF2 recruitment to the nucleus to trigger ferroptosis

**Fig. 5:**
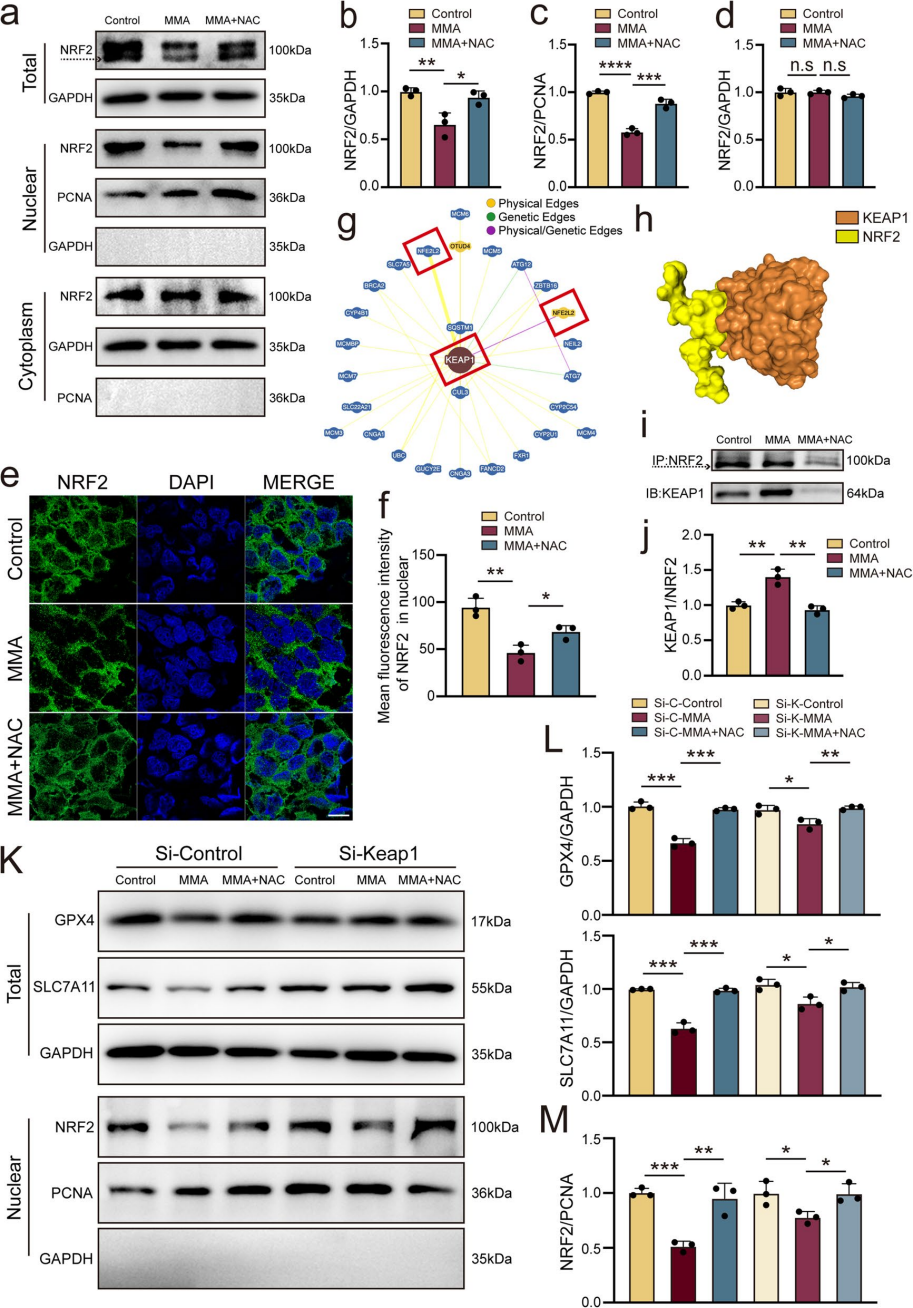
MMA inhibits NRF2 nuclear translocation. In vitro, AC16 cells were treated with MMA (17.5 mM) alone for 3 h or with (acetylcysteine) NAC (5 mM) for 2 h followed by MMA (17.5 mM) for 3 h. **a**-**d** NRF2 expression in the cytoplasm, nucleus and total protein of AC16 cells analysed by WB (*n* = 3/group). **e**, **f** Immunofluorescence analysis of the effect of MMA and NAC on NRF2 nucleus translocation in AC16 cells (*n* = 3/group) (Scale bar: 50 μm). **g**, **h** Binding model between KEAP1 and NRF2 were acquired by protein–protein predicted website BioGRID (http://thebiogrid.org) and HDOCK SEVER (http://hdock.phys.hust.edu.cn/). **i**, **j** Co-immunoprecipitation detected NRF2 and KEAP1 complex formation (*n* = 3/group). k-l The expression of ferroptosis-related proteins GPX4 and SLC7A11 were detected by western blot (*n* = 3/group). m Western blotting analysis of the protein level of NRF2 in AC16 cells in the nucleus (*n* = 3/group). Data are expressed as the mean ± standard deviation. *P < 0.05, **P < 0.01, ***P < 0.001, ****P < 0.0001 and *n.s*, not significant

Previous studies reported NRF2 as an important endogenous antioxidant [41, 42]. Moreover, its expression decreased gradually with the duration of reperfusion in mouse myocardial tissue of the I/R model, with the most significant difference observed at 24 h (Supplementary Fig. 7a, b). These results suggest that NRF2 plays an important role in I/R injury. Similarly, MMA impeded NRF2 expression in AC16 cells (Fig. 5a, b). MMA treatment for 3 h significantly reduced the nuclear ectopic expression of NRF2 in AC16 cells, whereas NAC treatment partially reversed this effect (Fig. 5a, c). However, cytoplasmic NRF2 did not change significantly after MMA treatment (Fig. 5a, d). Similarly, immunofluorescence staining showed less NRF2 in the nucleus after MMA treatment (Fig. 5e, f).

Based on the protein–protein prediction websites BioGRID (http://thebiogrid.org) and HDOCK SERVER (http://hdock.phys.hust.edu.cn/) (Fig. 5g, h), NRF2 might interact with KEAP1 in human and mouse. We further hypothesized that MMA might inhibit the entry of NRF2 into the nucleus by enhancing the binding of KEAP1 to NRF2. To test this hypothesis, co-immunoprecipitation analysis was performed with MMA-treated AC16 cells to reveal the endogenous interaction between KEAP1 and NRF2. Furthermore, MMA considerably increased the amount of NRF2-bound KEAP1 compared to that in the control group, suggesting that MMA might restrict the nuclear entry of NRF2 by promoting NRF2–KEAP1 binding, thereby inducing ferroptosis (Fig. 5i, j).

Next, KEAP1 siRNA was used to assess the contribution of KEAP1 to MMA-induced ferroptosis. Here, KEAP1 siRNA-2 effectively knocked down KEAP1 expression (Supplementary Fig. 7c, d); hence, KEAP1 siRNA-2 and control-siRNA were used in subsequent experiments. As expected, administration of MMA with control siRNA reduced GPX4 and SLC7A11 expression, whereas the inhibitory effect of MMA on GPX4 and SLC7A11 expression was partly reversed by KEAP1 silencing (Fig. 5k, l). Furthermore, KEAP1 silencing partially restored the ectopic nuclear expression of NRF2 upon MMA treatment (Fig. 5k, m). In summary, MMA inhibits the nuclear expression of NRF2 by enhancing KEAP1–NRF2 binding to activate ferroptosis.

### MMA-induced myocardial injury is alleviated by ferroptosis inhibitors

**Fig. 6:**
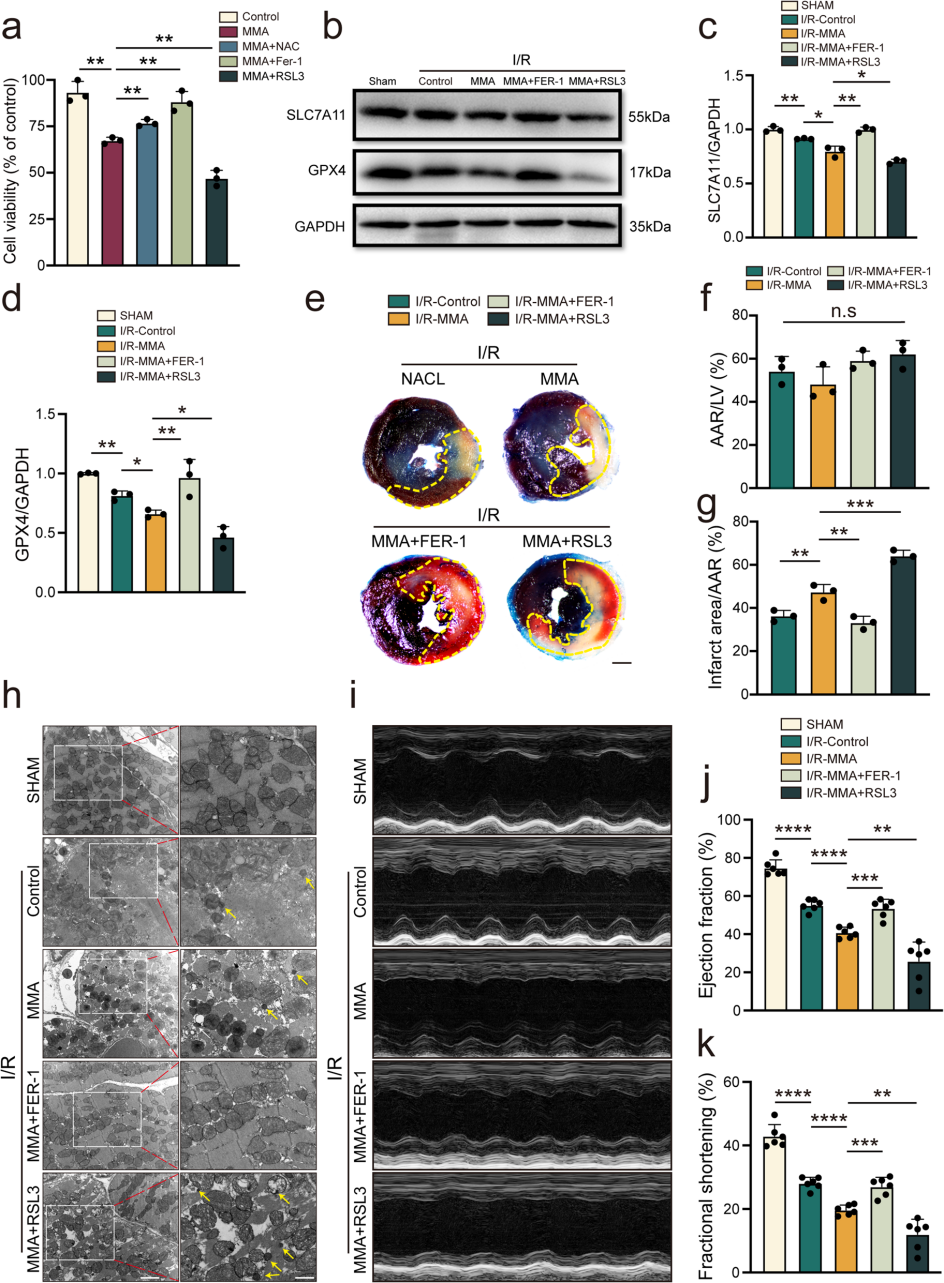
MMA-induced myocardial injury could be alleviated by ferroptosis inhibitor. In vitro, AC16 cells were treated with MMA (17.5 mM) alone for 3 h or with NAC (5 mM) for 2 h followed by MMA (17.5 mM) for 3 h. Otherwise, cells were treated by MMA (17.5 mM) with Ferrostatin-1 (Fer-1) (2 μM) or (1S,3R)-RSL3 (RSL3) (3 μM) for 3 h. In vivo, mice were assigned to the sham operation or myocardial I/R injury group. MMA (400 mg/kg/d) was administered seven days before the I/R injury. Meanwhile, NAC (300 mg/kg), Fer-1 (10 mg/kg​), and RSL3 (10 mg/kg​) were administered once in two days for three times before the I/R model (on day 2, 4, and 6 of the administration process). **a** Cell viability was detected by MTT assay after beinig administered with dimethyl sulfoxide (DMSO) as a control, MMA (15 mM), NAC (5 mM), Fer-1 (2 μM) or RSL3 (3 μM) (*n* = 3/group). **b**-**d** Western blot analysed of GPX4 and SLC7A11 in mice heart tissue (*n* = 3/group). **e**–**g** Representative images of heart sections with TTC/Evans staining and the ratios of the AAR to LV area and infarct area to AAR (*n* = 3/group). The area of AAR was marked with yellow dotted line. **h** TEM analysis of mitochondria in mice heart tissue. Yellow arrows indicate mitochondria (Scale bar: 1/2 μm). **i**-**k** Representative images of M-mode echocardiograms and quantitative analysis of EF and FS by echocardiography (*n* = 6/group). Data are expressed as the mean ± standard deviation. *P < 0.05, **P < 0.01, ***P < 0.001, ****P < 0.0001 and *n.s*, not significant

We then used RSL3 and Fer-1, a ferroptosis agonist and inhibitor, respectively, to investigate whether MMA treatment leads to enhanced ferroptosis. The MTT assay demonstrated that both RSL3 (3 μM) and Fer-1 (2 μM) did not affect cell viability (Supplementary Fig. 4a). Although Fer-1 tended to reduce LDH activity, this was not statistically significant compared to the control group. Similarly, in the RSL3 group, there was no significant difference in LDH activity compared to that in the control group (Supplementary Fig. 4b). Furthermore, neither RSL3 nor Fer-1 affected cardiac functions (Supplementary Fig. 4c–i). However, Fer-1 diminished the inhibitory effect of MMA on cellular activity, which was further aggravated by RSL3 (Fig. 6a).

Western blotting was then used to examine the effects of FER-1 and RSL3 in MMA-stimulated I/R-model mice by measuring SLC7A11 and GPX4 expression. SLC7A11 and GPX4 levels in the I/R-MMA + FER-1 group were significantly higher than those in the I/R-MMA group, whereas RSL3 pretreatment prevented SLC7A11 and GPX4 production (Fig. 6b–d). The inhibition of ferroptosis, mediated by FER-1, profoundly reduced the infarct size but had no notable effect on the AAR. In contrast, RSL3 increased the infarct area, indicating that the inhibition and activation of ferroptosis might be related to myocardial injury (Fig. 6e–g). Furthermore, through TEM, smaller mitochondria in the heart tissue of the I/R-MMA + RSL3 group were more evident than the IR-MMA group. In contrast, these alterations were largely absent in the heart samples treated with FER-1 (Fig. 6h). Consistently, I/R-induced cardiac dysfunction was further enhanced in RSL3-treated mice compared to I/R-MMA mice, as indicated by a further decrease in EF and FS values. However, cardiac dysfunction was partially relieved in the FER-1-treated mice (Figs. 6i–k).

## Discussion

AMI is a severe and potentially life-threatening disease among the most common causes of mortality worldwide. Here, we observed that plasma MMA concentrations are increased in patients with AMI and in mouse myocardial tissue following I/R (45 min of ischemia and 24 h of reperfusion). Elevated MMA levels induced oxidative stress in cardiomyocytes by increasing the expression of NOX2 and NOX4. Activated oxidative stress then triggered ferroptosis, which was further demonstrated based on the downregulation of SLC7A11 and GPX4 expression in response to MMA treatment. Meanwhile, the inhibition of oxidative stress and ferroptosis blocked the myocardial damage caused by MMA, indicating that myocardial injury in the I/R model was closely related to MMA-driven ferroptosis. Mechanistically, MMA-induced ferroptosis was attributed to the reduced ectopic nuclear expression of NRF2 through increased NRF2–KEAP1 binding.

We previously found that individuals with high MMA levels have an increased risk of all-cause and cardiovascular mortality, suggesting that it might represent a prognostic biomarker in adults [32]. In this study, we found that MMA concentrations are increased in patients with AMI and in AC16 cells stimulated by H_2_O_2_. In addition, MMA levels were elevated in the myocardial tissue of I/R-model mice. MMA treatment led to increased myocardial injury in mice, which manifested as decreased myocardial function and increased infarct size. These results indicate that the concentration of MMA increases during myocardial ischemia and hypoxia. Subsequently, elevated MMA levels exacerbate myocardial injury during perfusion.

Previous clinical studies have reported that MMA, a metabolite derived from the mitochondria, can induce oxidative stress in patients of different ages [43]. During I/R, mitochondrial-induced oxidative stress bursts lead to myocardial cell damage [44]. Moreover, recent studies have shown that mitochondrial dynamics are critical role in I/R injury [45]. In this study, we found that the accumulation of MMA inhibited energy production in AC16 cells and activated mitochondrial fission via the upregulation of DRP1, MFF, and FIS1 expression but had minor effect on mitochondrial fusion. MMA also decreased the mitochondrial membrane potential and increased mitochondria-derived ROS, as evidenced by the JC-1 staining and the MitoSOX probe. These findings suggest that MMA impairs mitochondrial dynamics and induces mitochondria-derived oxidative stress.

NADPH oxidase is an important pro-oxidase that produces ROS and comprises seven distinct subfamilies, including NOX1–5 and the dual oxidases (DUOX1 and DUOX2) [46]. ROS can maintain cellular functions by regulating intracellular signaling and the redox balance. However, excessive ROS levels can cause oxidative damage to biomacromolecules and organelles [47]. Our results revealed elevated NOX2 and NOX4 expression in vitro and in vivo following MMA treatment, indicating that MMA leads to excessive ROS production. We also examined the effects of pretreatment with NAC, a commonly used oxidative stress inhibitor, on cardiomyocytes [48]. Our data demonstrated that NAC relieved MMA-treated cardiomyocyte injury by suppressing NOX2 and NOX4 overexpression in vivo and in vitro, as compared to levels in the MMA group. These findings suggest that oxidative stress is a critical contributor to MMA-induced myocardial damage.

Ferroptosis is a ROS-dependent cell death mechanism with two main characteristics, specifically iron uploading and lipid peroxidation [17]. Lipid hydroperoxides (LOOHs) are produced through ferroptosis-related lipid peroxidation, triggered by interactions between polyunsaturated fatty acids and the free radicals found in biofilms [12, 49]. LOOHs are then oxidized to MDA and 4HNE, two common end products of lipid peroxidation. MMA treatment significantly increased MDA levels and Liperfluo fluorescence intensity in AC16 cells, indicating marked lipid peroxidation. The cystine/glutamate antiporter (system xc-) is an amino acid transporter located on the mammalian cell membrane and is composed of a light chain (SLC7A11) and heavy chains (solute carrier family 3 members 2, SLC3A2), the activity of which is primarily controlled by SLC7A11 [50]. Under normal metabolic conditions, extracellular cystine is transported into the cell via system xc-. Intracellularly, cystine is reduced to cysteine, which is an essential raw material for the synthesis of GSH, an important cellular antioxidant that exists in dynamic equilibrium with oxidized GPX4 [50, 51]. The primary mechanism facilitating ferroptosis is system xc-/GSH/GPX4 antioxidant system breakdown, and the decreased expression of its key proteins SLC7A11 and GPX4 reduces the antioxidant capacity of cells [19, 52]. Previous studies have shown that I/R decreases the expression of GPX4 and SLC7A11 [18, 19], which is consistent with our results. Interestingly, GPX4 and SLC7A11 levels were further reduced in the I/R-MMA group compared to the I/R-Control group, suggesting that the increase in MMA in the I/R model might comprise an important mechanism associated with ferroptosis in cardiomyocytes. Moreover, ferroptosis is often manifested by mitochondrial abnormalities, such as condensation or swelling, a decrease in or the disappearance of cristae, and damaged membranes [12, 40, 52]. TEM analysis showed that MMA caused mitochondrial shrinkage and loss of the mitochondrial cristae, suggesting mitochondrial dysfunction and ferroptosis activation.

Evidence suggests that FER-1 decreases ferroptosis by increasing GPX4 expression [53]. Our study also showed upregulated GPX4 and SLC7A11 improved cardiac function and reduced myocardial infarction size in the I/R-MMA + FER-1 group, as compared to the I/R-MMA group. In contrast, RSL3, an activator of ferroptosis, reduced GPX4 and SLC7A11 expression, suggestive of ferroptosis activation, which led to a decrease in cardiac function and an increase in the myocardial infarct size. A recent study reported that RSL3 inhibits GPX4 expression and activates ferroptosis, which is consistent with our findings [54]. NAC reversed the inhibitory effect on GPX4 and SLC7A11 expression in MMA-treated cells while also reversing the effects on GSH levels mediated by MMA treatment. These results suggest that the inhibition of oxidative stress alleviates MMA-induced cell ferroptosis.

NRF2 is one of the main transcription factors involved in the cellular antioxidant response. The proteasome usually destroys NRF2 after interacting with its regulator KEAP1, which is subsequently ubiquitinated. However, promoting the expression of NRF2 during myocardial I/R injury enhances the transcription of SLC7A11 and GPX4, thereby inhibiting cardiomyocyte ferroptosis [55]. Our study found that GPX4 and SLC7A11 expression was markedly suppressed, similar to NRF2 levels, in AC16 cells after MMA treatment. Meanwhile, the phosphorylated form of NRF2 can be released from KEAP1 and translocates to the nucleus, thereby promoting the transcription of downstream target genes [56]. Indeed, our results showed that MMA treatment markedly reduced ectopic NRF2 nuclear expression and significantly boosted NRF2–KEAP1 binding. Hence, MMA might activate ferroptosis in cardiomyocytes by inhibiting ectopic NRF2 nuclear expression and enhancing NRF2–KEAP1 binding.

This study has certain limitations. First, the specific molecular binding domains between KEAP1 and NRF2 were not identified. Second, though substantial results were derived from in vitro and in vivo models, further investigations are required for the clinical translation of our results.

## Conclusion

**Fig. 7:**
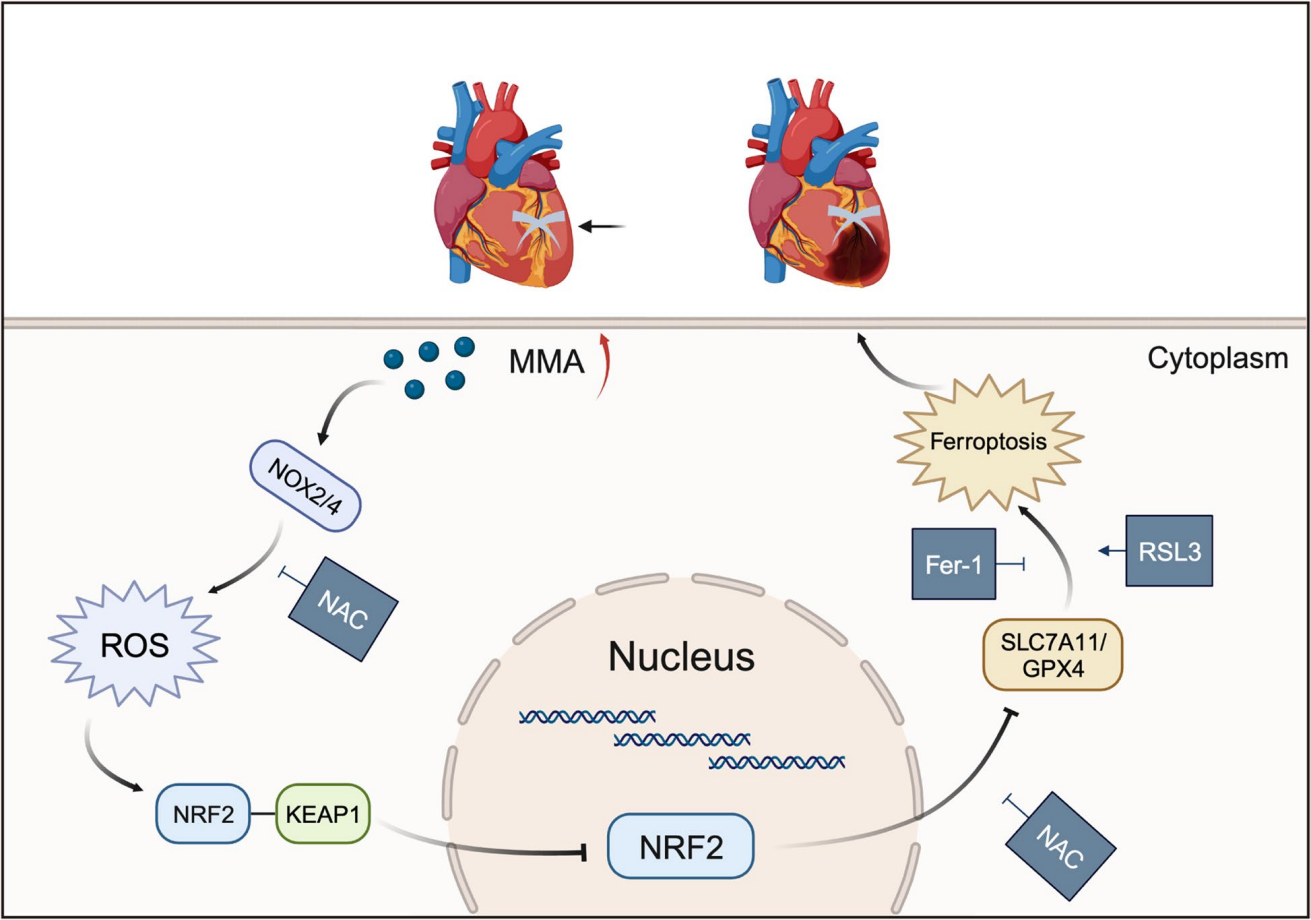
Mechanism of myocardial injury caused by MMA

In conclusion, our study revealed, for the first time, that mitochondrial-derived metabolite MMA plays a pivotal role in the pathogenesis of cardiomyocyte death under I/R stress. MMA-related myocardial injury is triggered by the induction of oxidative stress and ferroptosis in cardiomyocytes. Additionally, MMA strengthens the interaction between NRF2 and KEAP1 to inhibit the ectopic nuclear distribution of NRF2 (Fig. 7), which is of great significance for studying the progression of I/R injury. Our study provides novel insights into the mechanisms underlying MMA-induced myocardial injury in an I/R model.

### Supplementary Information

**Additional file 1.**

**Additional file 2: Supplementary Table 1.** Baseline characteristics of participants. **Supplementary Table 2.** siRNAs for transfection. **Supplementary Table 3.** Antibodies. **Supplementary Table 4.** Primers. **Supplementary Fig. 1.** MMA elevated in conditions of ischemia-reperfusion. **Supplementary Fig. 2.** MMA affected mitochondrial dynamics. **Supplementary Fig. 3.** MMA and H_2_O_2_ caused cell damage. **Supplementary Fig. 4.** NAC, Fer-1 and RSL3 did not affect myocardial function. **Supplementary Fig. 5.** MMA treatment triggered programmed cell death. **Supplementary Fig. 6.** MitoTEMPO reversed the lipid peroxidation and cell damage caused by MMA.

## Data Availability

Upon reasonable request, the corresponding author will provide data to support the results of this study.

## Abbreviations

AAR: Area at risk
DCFH-DA: 2,7-Dichlorodi-hydrofluorescein diacetate
DHE: Dihydroethidium
GSH: Glutathione
MDA: Malondialdehyde
NAC: N-acetylcysteine
KEAP1: Kelch-like ECH-associated protein 1
NRF2: Nuclear factor E2-related factor 2
MMA: Methylmalonic acid
AMI: Acute myocardial infarction
I/R: Ischemia–reperfusion
ROS: Reactive oxygen species
H/R: Hypoxia/reoxygenation
EF: Ejection fraction
FS: Fractional shortening
GPX4: Glutathione peroxidase 4
SLC7A11: Solute carrier family 7 member 11
Fer-1: Ferrostatin-1
RSL3: (1S,3R)-RSL3

## References

[1] DeFilippis AP, Nasir K, Blaha MJ (2019). Myocardial infarction as a clinical end point in research. Circ Res.

[2] Tamis-Holland JE, Jneid H, Reynolds HR, Agewall S, Brilakis ES, Brown TM, Lerman A, Cushman M, Kumbhani DJ, Arslanian-Engoren C, Bolger AF, Beltrame JF (2019). Contemporary diagnosis and management of patients with myocardial infarction in the absence of obstructive coronary artery disease: a scientific statement from the american heart association. Circulation.

[3] Hu SY, Zhang Y, Zhu PJ, Zhou H, Chen YD (2017). Liraglutide directly protects cardiomyocytes against reperfusion injury possibly via modulation of intracellular calcium homeostasis. J Geriatr Cardiol.

[4] Zhou H, Hu S, Jin Q, Shi C, Zhang Y, Zhu P, Ma Q, Tian F, Chen Y (2017). Mff-Dependent Mitochondrial Fission Contributes to the Pathogenesis of Cardiac Microvasculature Ischemia/Reperfusion Injury via Induction of mROS-Mediated Cardiolipin Oxidation and HK2/VDAC1 Disassociation-Involved mPTP Opening. J Am Heart Assoc.

[5] Kloner RA, Ganote CE, Jennings RB (1974). The "no-reflow" phenomenon after temporary coronary occlusion in the dog. J Clin Invest.

[6] Zhou H, Wang S, Zhu P, Hu S, Chen Y, Ren J (2018). Empagliflozin rescues diabetic myocardial microvascular injury via AMPK-mediated inhibition of mitochondrial fission. Redox Biol.

[7] Zweier JL, Talukder MA (2006). The role of oxidants and free radicals in reperfusion injury. Cardiovasc Res.

[8] Galang N, Sasaki H, Maulik N (2000). Apoptotic cell death during ischemia/reperfusion and its attenuation by antioxidant therapy. Toxicology.

[9] Werns SWSM, Lucchesi BR (1986). Free radicals and myocardial injury: pharmacologic implications. Circulation Circulation.

[10] Kurian GA, Rajagopal R, Vedantham S, Rajesh M (2016). The role of oxidative stress in myocardial ischemia and reperfusion injury and remodeling: revisited. Oxid Med Cell Longev.

[11] Moens AL, Claeys MJ, Timmermans JP, Vrints CJ (2005). Myocardial ischemia/reperfusion-injury, a clinical view on a complex pathophysiological process. Int J Cardiol.

[12] Dixon SJ, Lemberg KM, Lamprecht MR, Skouta R, Zaitsev EM, Gleason CE, Patel DN, Bauer AJ, Cantley AM, Yang WS, Morrison B, Stockwell BR (2012). Ferroptosis: an iron-dependent form of nonapoptotic cell death. Cell.

[13] Wu X, Li Y, Zhang S, Zhou X (2021). Ferroptosis as a novel therapeutic target for cardiovascular disease. Theranostics.

[14] Anandhan A, Dodson M, Schmidlin C, Liu P, Zhang D (2020). Breakdown of an Ironclad Defense System: The Critical Role of NRF2 in Mediating Ferroptosis. Cell Chem Biol.

[15] Capelletti M, Manceau H, Puy H, Peoc'h K (2020). Ferroptosis in Liver Diseases: An Overview. Int J Mol Sci.

[16] Ravingerová T, Kindernay L, Barteková M, Ferko M, Adameová A, Zohdi V, Bernátová I, Ferenczyová K, Lazou A (2020). The molecular mechanisms of iron metabolism and its role in cardiac dysfunction and cardioprotection. Int J Mol Sci.

[17] Tang D, Chen X, Kang R, Kroemer G (2021). Ferroptosis: molecular mechanisms and health implications. Cell Res.

[18] Feng Y, Madungwe NB, Imam Aliagan AD, Tombo N, Bopassa JC (2019). Liproxstatin-1 protects the mouse myocardium against ischemia/reperfusion injury by decreasing VDAC1 levels and restoring GPX4 levels. Biochem Biophys Res Commun.

[19] Ma S, Sun L, Wu W, Wu J, Sun Z, Ren J (2020). USP22 Protects Against Myocardial Ischemia-Reperfusion Injury via the SIRT1-p53/SLC7A11-Dependent Inhibition of Ferroptosis-Induced Cardiomyocyte Death. Front Physiol.

[20] Moi P, Chan K, Asunis I, Cao A, Kan Y (1994). Isolation of NF-E2-related factor 2 (Nrf2), a NF-E2-like basic leucine zipper transcriptional activator that binds to the tandem NF-E2/AP1 repeat of the beta-globin locus control region. Proc Natl Acad Sci USA.

[21] Yu X, Kensler T (2005). Nrf2 as a target for cancer chemoprevention. Mutat Res/Fundamental and Molecular Mechanisms of Mutagenesis.

[22] Lee J, Johnson J (2004). An important role of Nrf2-ARE pathway in the cellular defense mechanism. J Biochem Mol Biol.

[23] Hwang JW, Park JH, Park BW, Kim H, Kim JJ, Sim WS, Mishchenko NP, Fedoreyev SA, Vasileva EA, Ban K, Park HJ, Baek SH (2021). Histochrome Attenuates Myocardial Ischemia-Reperfusion Injury by Inhibiting Ferroptosis-Induced Cardiomyocyte Death. Antioxidants (Basel).

[24] Lin J, Yang K, Ting P, Luo Y, Lin D, Wang Y, Chang J (2021). Gossypol Acetic Acid Attenuates Cardiac Ischemia/Reperfusion Injury in Rats via an Antiferroptotic Mechanism. Biomolecules.

[25] Green R, Allen LH, Bjørke-Monsen AL, Brito A, Guéant JL, Miller JW, Molloy AM, Nexo E, Stabler S, Toh BH, Ueland PM, Yajnik C (2017). Vitamin B(12) deficiency. Nat Rev Dis Primers.

[26] Hannibal L, Lysne V, Bjørke-Monsen A, Behringer S, Grünert S, Spiekerkoetter U, Jacobsen D, Blom H (2016). Biomarkers and Algorithms for the Diagnosis of Vitamin B12 Deficiency. Front Mol Biosci.

[27] Guo J, Liu X, Wang Z, Lu R, Liu Y, Zhang Y, Tian W, Fang S, Wang S, Yu B (2023). Methylmalonic acid, vitamin B12, and mortality risk in patients with preexisting coronary heart disease: a prospective cohort study. Nutr J.

[28] Wang S, Wang Y, Wan X, Guo J, Zhang Y, Tian M, Fang S, Yu B (2022). Cobalamin Intake and Related Biomarkers: Examining Associations With Mortality Risk Among Adults With Type 2 Diabetes in NHANES. Diabetes Care.

[29] Melo DR, Mirandola SR, Assunção NA, Castilho RF (2012). Methylmalonate impairs mitochondrial respiration supported by NADH-linked substrates: involvement of mitochondrial glutamate metabolism. J Neurosci Res.

[30] Stepien KM, Heaton R, Rankin S, Murphy A, Bentley J, Sexton D, Hargreaves IP (2017). Evidence of Oxidative Stress and Secondary Mitochondrial Dysfunction in Metabolic and Non-Metabolic Disorders. J Clin Med.

[31] Wajner M, Coelho JC (1997). Neurological dysfunction in methylmalonic acidaemia is probably related to the inhibitory effect of methylmalonate on brain energy production. J Inherit Metab Dis.

[32] Wang S, Liu Y, Liu J, Tian W, Zhang X, Cai H, Fang S, Yu B (2020). Mitochondria-derived methylmalonic acid, a surrogate biomarker of mitochondrial dysfunction and oxidative stress, predicts all-cause and cardiovascular mortality in the general population. Redox Biol.

[33] Wang S, Chen K, Wang Y, Wang Z, Li Z, Guo J, Chen J, Liu W, Guo X, Yan G, Liang C, Yu H, Fang S, Yu B (2023). Cardiac-targeted delivery of nuclear receptor RORα via ultrasound targeted microbubble destruction optimizes the benefits of regular dose of melatonin on sepsis-induced cardiomyopathy. Biomaterials research.

[34] Luciani A, Schumann A, Berquez M, Chen Z, Nieri D, Failli M, Debaix H, Festa BP, Tokonami N, Raimondi A, Cremonesi A, Carrella D, Forny P, Kölker S, Diomedi Camassei F, Diaz F, Moraes CT, Di Bernardo D, Baumgartner MR, Devuyst O (2020). Impaired mitophagy links mitochondrial disease to epithelial stress in methylmalonyl-CoA mutase deficiency. Nat Commun.

[35] Stockwell BR (2022). Ferroptosis turns 10: Emerging mechanisms, physiological functions, and therapeutic applications. Cell.

[36] Gryzik M, Asperti M, Denardo A, Arosio P, Poli M (2021). NCOA4-mediated ferritinophagy promotes ferroptosis induced by erastin, but not by RSL3 in HeLa cells. Biochim Biophys Acta Mol Cell Res.

[37] Liu X, Xia S, Zhang Z, Wu H, Lieberman J (2021). Channelling inflammation: gasdermins in physiology and disease. Nat Rev Drug Discov.

[38] Varfolomeev EE, Ashkenazi A (2004). Tumor necrosis factor: an apoptosis JuNKie?. Cell.

[39] Xiong S, Mu T, Wang G, Jiang X (2014). Mitochondria-mediated apoptosis in mammals. Protein Cell.

[40] Friedmann Angeli JP, Schneider M, Proneth B, Tyurina YY, Tyurin VA, Hammond VJ, Herbach N, Aichler M, Walch A, Eggenhofer E, Basavarajappa D, Radmark O, Kobayashi S, Seibt T, Beck H, Neff F, Esposito I, Wanke R, Forster H, Yefremova O, Heinrichmeyer M, Bornkamm GW, Geissler EK, Thomas SB, Stockwell BR, O'Donnell VB, Kagan VE, Schick JA, Conrad M (2014). Inactivation of the ferroptosis regulator Gpx4 triggers acute renal failure in mice. Nat Cell Biol.

[41] Xu S, Wu B, Zhong B, Lin L, Ding Y, Jin X, Huang Z, Lin M, Wu H, Xu D (2021). Naringenin alleviates myocardial ischemia/reperfusion injury by regulating the nuclear factor-erythroid factor 2-related factor 2 (Nrf2) /System xc-/ glutathione peroxidase 4 (GPX4) axis to inhibit ferroptosis. Bioengineered.

[42] Zhang X, Yu Y, Lei H, Cai Y, Shen J, Zhu P, He Q, Zhao M (2020). The Nrf-2/HO-1 Signaling Axis: a ray of hope in cardiovascular diseases. Cardiol Res Pract.

[43] Solomon LR (2015). Functional cobalamin (vitamin B12) deficiency: role of advanced age and disorders associated with increased oxidative stress. Eur J Clin Nutr.

[44] Zhou H, Li D, Shi C, Xin T, Yang J, Zhou Y, Hu S, Tian F, Wang J, Chen Y (2015). Effects of Exendin-4 on bone marrow mesenchymal stem cell proliferation, migration and apoptosis in vitro. Sci Rep.

[45] Jin Q, Li R, Hu N, Xin T, Zhu P, Hu S, Ma S, Zhu H, Ren J, Zhou H (2018). DUSP1 alleviates cardiac ischemia/reperfusion injury by suppressing the Mff-required mitochondrial fission and Bnip3-related mitophagy via the JNK pathways. Redox Biol.

[46] Bedard K, Krause KH (2007). The NOX family of ROS-generating NADPH oxidases: physiology and pathophysiology. Physiol Rev.

[47] Pajares M, Cuadrado A, Engedal N, Jirsova Z, Cahova M (2018). The Role of Free Radicals in Autophagy Regulation: Implications for Ageing. Oxid Med Cell Longev.

[48] Farr SAPH, Dogrukol-Ak D, Drake J, Banks WA, Eyerman E, Butterfield DA, Morley JE (2003). The antioxidants alpha-lipoic acid and N-acetylcysteine reverse memory impairment and brain oxidative stress in aged SAMP8 mice. J Neurochem.

[49] Ayala A, Munoz MF, Arguelles S (2014). Lipid peroxidation: production, metabolism, and signaling mechanisms of malondialdehyde and 4-hydroxy-2-nonenal. Oxid Med Cell Longev.

[50] Leclercq K, Liefferinge JV, Albertini G, Neveux M, Dardenne S, Mairet-Coello G, Vandenplas C, Deprez T, Chong SA, Foerch P, Bentea E, Sato H, Maher P, Massie A, Smolders I, Kaminski RM (2019). Anticonvulsant and antiepileptogenic effects of system xc- inactivation in chronic epilepsy models. Epilepsia.

[51] Bridges RJ, Natale NR, Patel SA (2012). System xc(-) cystine/glutamate antiporter: an update on molecular pharmacology and roles within the CNS. Br J Pharmacol.

[52] Chen X, Kang R, Kroemer G, Tang D (2021). Organelle-specific regulation of ferroptosis. Cell Death Differ.

[53] Liu P, Feng Y, Li H, Chen X, Wang G, Xu S, Li Y, Zhao L (2020). Ferrostatin-1 alleviates lipopolysaccharide-induced acute lung injury via inhibiting ferroptosis. Cell Mol Biol Lett.

[54] Shin D, Kim EH, Lee J, Roh JL (2018). Nrf2 inhibition reverses resistance to GPX4 inhibitor-induced ferroptosis in head and neck cancer. Free Radic Biol Med.

[55] Yang WS, Kim KJ, Gaschler MM, Patel M, Shchepinov MS, Stockwell BR (2016). Peroxidation of polyunsaturated fatty acids by lipoxygenases drives ferroptosis. Proc Natl Acad Sci U S A.

[56] Lu J, Zhao Y, Liu M, Lu J, Guan S (2021). Toward improved human health: Nrf2 plays a critical role in regulating ferroptosis. Food Funct.

