# Supplementary material for: Mitochondria-derived methylmalonic acid aggravates ischemia–reperfusion injury by activating reactive oxygen species-dependent ferroptosis
Source: Cell Commun Signal. 2024 Jan 18;22:53. doi: 10.1186/s12964-024-01479-z (PMC10797736; doi:10.1186/s12964-024-01479-z)

Figure 2k- NOX2/GAPDH

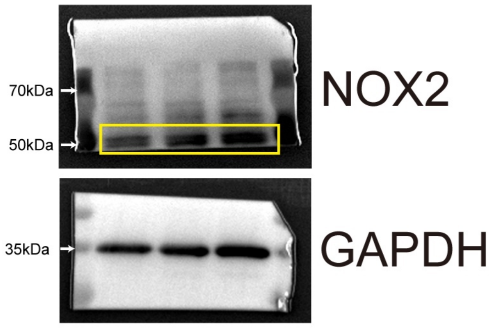

Figure 2k- NOX4/GAPDH

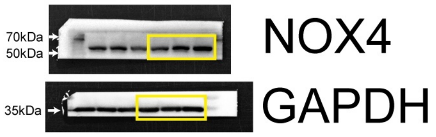

Figure 3g- NOX2/GAPDH

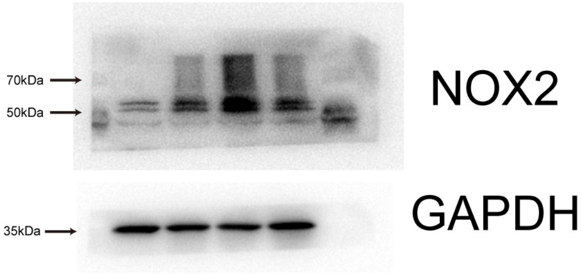

Figure 3g- NOX4/GAPDH

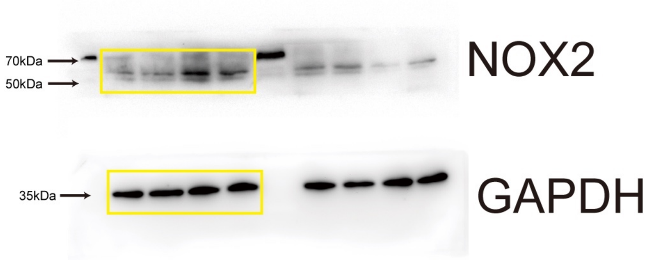

Figure 3i- NOX2/GAPDH

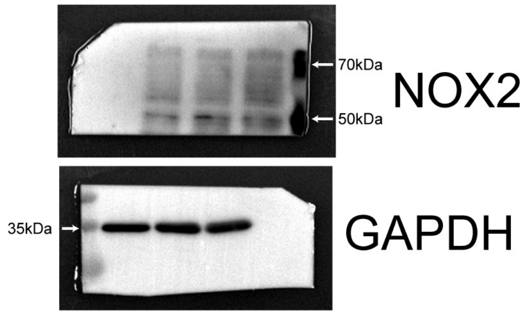

Figure 3i- NOX4

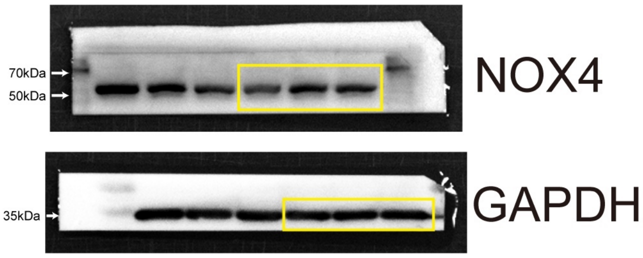

Figure 4e- GPX4/GAPDH

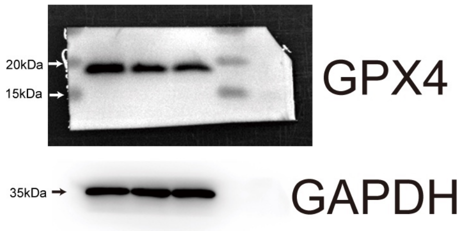

Figure 4e- SLC7A11/GAPDH

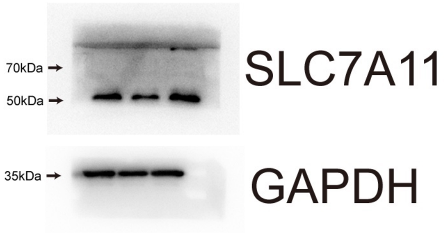

Figure 4g- SLC7A11/GAPDH

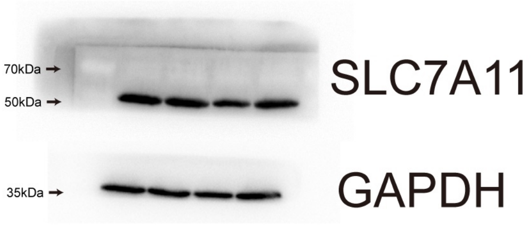

Figure 4g- GPX4/GAPDH

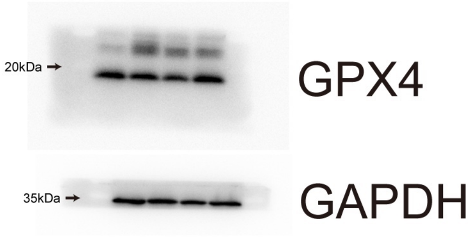

Figure 5a- NRF2/GAPDH-Total

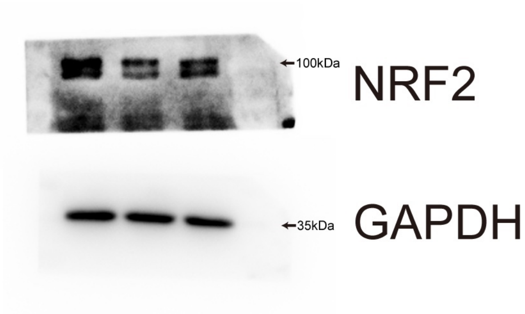

Figure 5a- NRF2/PCNA/GAPDH-Nuclear

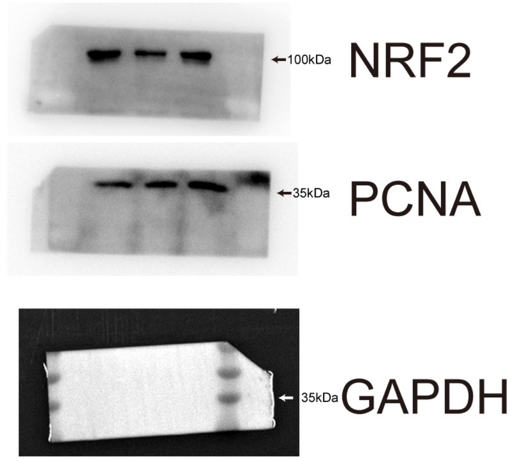

Figure 5a- NRF2/PCNA/GAPDH-Cytoplasm

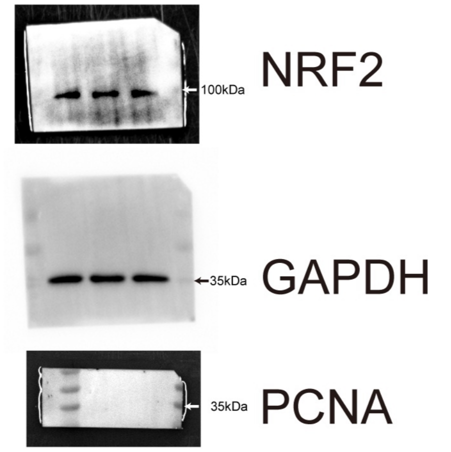

Figure 5i- NRF2/KEAP1

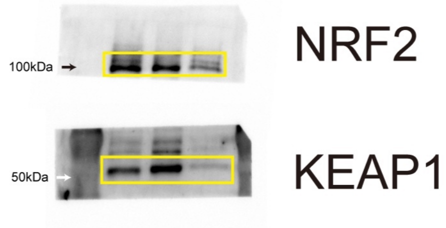

Figure 5K- GPX4/GAPDH-Total

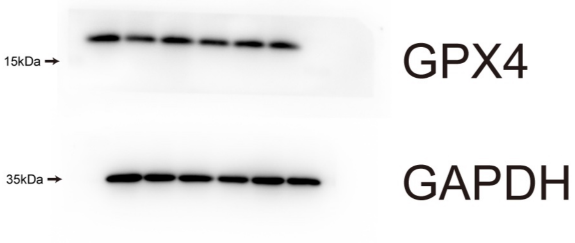

Figure 5K- SLC7A11/GAPDH-Total

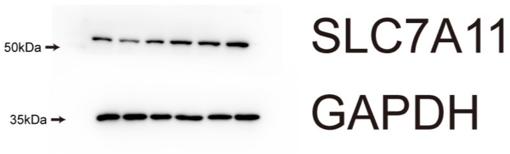

Figure 5K- NRF2/PCNA/GAPDH-Nuclear

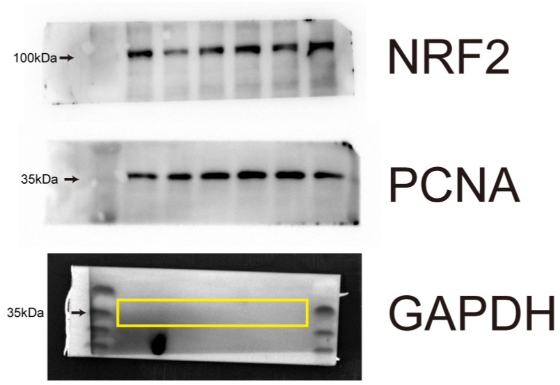

Figure 6b- GPX4/GAPDH

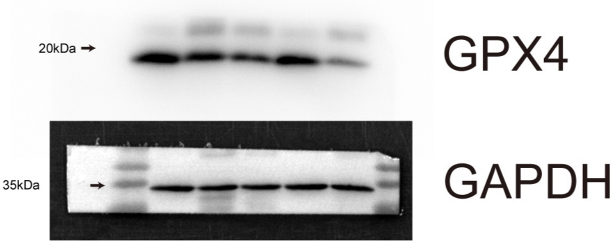

Figure 6b- SLC7A11/GAPDH

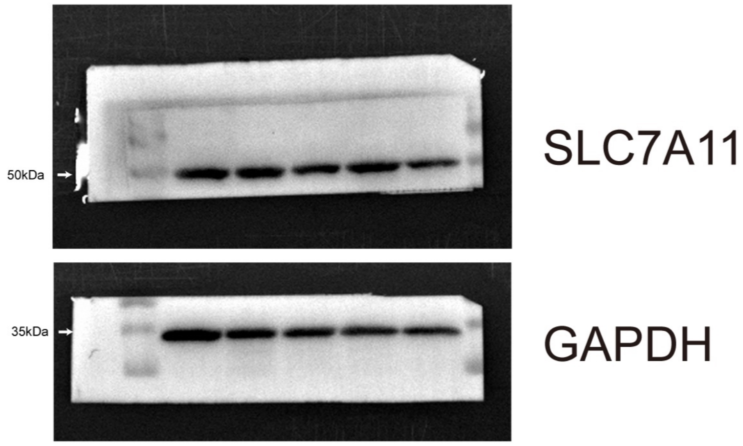

Supplementary Figure 1e MMUT/GAPDH

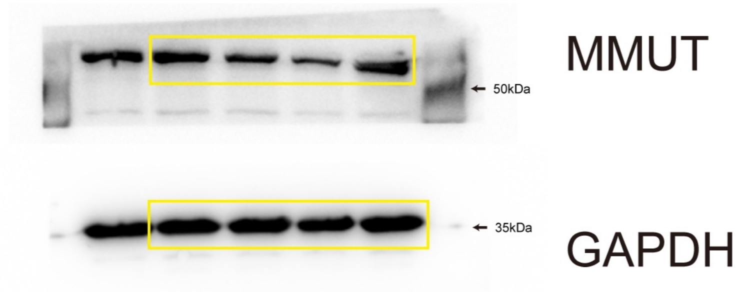

Supplementary Figure 2i DRP1/GAPDH

Supplementary Figure 2e DRP1/GAPDH

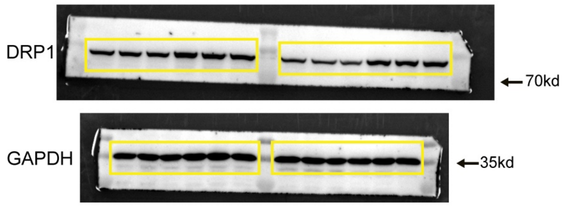

Supplementary Figure 2i MFN1/GAPDH

Supplementary Figure 2e MFN1/GAPDH

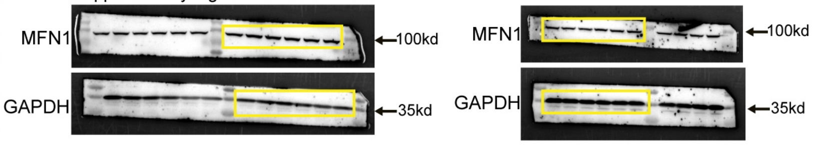

Supplementary Figure 7b- NRF2/GAPDH

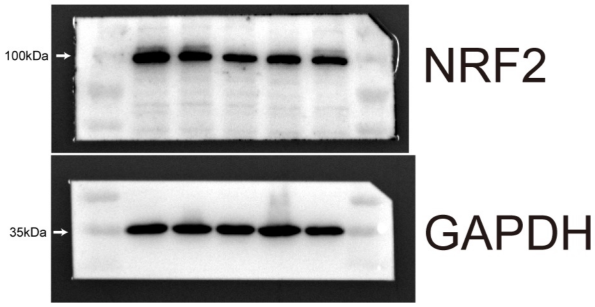

Supplementary Figure 7C- KEAP1/GAPDH

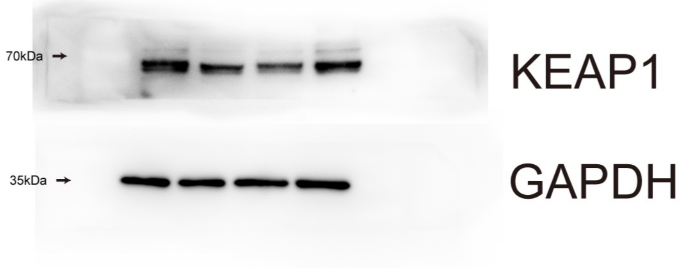

Supplement: Supplementary file 1 — Additional file 1. [file 12964_2024_1479_MOESM1_ESM.pdf]
