# Supplementary material for: Mitochondria-derived methylmalonic acid aggravates ischemia–reperfusion injury by activating reactive oxygen species-dependent ferroptosis
Source: Cell Commun Signal. 2024 Jan 18;22:53. doi: 10.1186/s12964-024-01479-z (PMC10797736; doi:10.1186/s12964-024-01479-z)
Supplement: Supplementary file 2 — Additional file 2: Supplementary Table 1. Baseline characteristics of participants. Supplementary Table 2. siRNAs for transfection. Supplementary Table 3. Antibodies. Supplementary Table 4. Primers. Supplementary Fig. 1. MMA elevated in conditions of ischemia-reperfusion. Supplementary Fig. 2. MMA affected mitochondrial dynamics. Supplementary Fig. 3. MMA and H2O2 caused cell damage. Supplementary Fig. 4. NAC, Fer-1 and RSL3 did not affect myocardial function. Supplementary Fig. 5. MMA treatment triggered programmed cell death. Supplementary Fig. 6. MitoTEMPO reversed the lipid peroxidation and cell damage caused by MMA. [file 12964_2024_1479_MOESM2_ESM.docx]

**Supplementary Table. 1** **Baseline characteristics of participants**

| **Variables** | **Overall (n=65)** | **Control (n=15)** | **AMI (n=50)** | **p trend** |
| --- | --- | --- | --- | --- |
| Age,y | 60.35±9.18 | 60.13±9.26 | 60.42±9.15 | 0.917 |
| Sex, % |  |  |  | 0.004 |
| Female | 41.54 | 73.33 | 32.00 |  |
| Male | 58.46 | 26.67 | 68.00 |  |
| BMI, kg/m^2^ | 24.51±4.06 | 24.74±5.27 | 24.45±3.61 | 0.392 |
| Smoking (current + ex), % | 56.92 | 33.33 | 64.00 | 0.035 |
| Alcohol (current + ex), % | 26.15 | 13.33 | 30.00 | 0.198 |
| Hypertension, % | 50.77 | 26.67 | 58.00 | 0.033 |
| Diabetes, % | 10.77 | 6.67 | 12.00 | 0.559 |
| cTnI, ug/L | 16.00±49.07 | 0.10±0.36 | 20.54±54.79 | 0.026 |
| TC, mmol/L | 4.53±1.04 | 4.14±0.86 | 4.64±1.06 | 0.238 |
| TG, mmol/L | 1.49±0.79 | 1.66±0.57 | 1.44±0.84 | 0.032 |
| HDL-C, mmol/L | 1.34±0.36 | 1.44±0.42 | 1.31±0.34 | 0.792 |
| LDL-C, mmol/L | 2.74±0.92 | 2.37±0.93 | 2.84±0.89 | 0.158 |
| hs-CRP, mg/L | 6.70±5.25 | 2.09±3.36 | 7.90±4.98 | <0.001 |

AMI, acute myocardial infarction; BMI, body mass index; cTNI, cardiac troponin I; hs-CRP, high-sensitivity C reactive protein; TC, totalcholesterol; TG, triglyceride.

**Supplementary Table. 2 siRNAs for transfection**

| **siRNA** | Sense (5’-3’) | Antisense (5’-3’) |
| --- | --- | --- |
| Negative control | UUCUCCGAACGUGUCACGUTT | ACGUGACACGUUCGGAGAATT |
| KEAP1-human-280 | GCUACACCCUGGAGGAUCATT | UGAUCCUCCAGGGUGUAGCTT |
| KEAP1-human-757 | GGGAGGUGGCCAAGCAAGATT | UCUUGCUUGGCCACCUCCCTT |
| KEAP1-human-1138 | UCAGCUACCUGGAGGCUUATT | UAAGCCUCCAGGUAGCUGATT |
| MMUT-human-787 | UGGAGAAAGUAAAGAGAUATT | UAUCUCUUUACUUUCUCCATT |
| MMUT-human-1153 | GAUGAGAGCUGGUAGAAGATT | UCUUCUACCAGCUCUCAUCTT |
| MMUT-human-1963 | UGGAGAAAGUAAAGAGAUATT | UAUCUCUUUACUUUCUCCATT |

**Supplementary Table. 3 Antibodies**

| **Antibodies** | **Source** | **Identifier** |
| --- | --- | --- |
| GAPDH | ZSGB-BIO | Cat#: ta-08 |
| PCNA | Proteintech | Cat#: 60097-1-lg |
| NOX4 | Proteintech | Cat#: 14347-1-AP |
| NOX2 | Proteintech | Cat#: 19013-1-AP |
| GPX4 | Abcam | Cat#: ab125066 |
| SLC7A11 | Abcam | Cat#: ab175186 |
| NRF2 | Abcam | Cat#: ab62352 |
| KEAP1 | Proteintech | Cat#: 10503-2-AP |
| DRP1 | Abcam | Cat#: ab184247 |
| MFN1 | Proteintech | Cat#: 13798-1-AP |

**Supplementary Table. 4 Primers**

| Gene | Forward primers (5’-3’) | Reverse primers (5’-3’) |
| --- | --- | --- |
| ACSL1 | GGAACTACAGGCAACCCCAA | ATCATCTGGGCAAGGATTGACT |
| ACSL4 | CCTTTTTGCGAGCTTTCCGAG | CAGCCAAGGCAGTTCAATCTT |
| LPCAT3 | AGCCTTAACAAGTTGGCGAC | GAGTGGTAGAGCTGGTTTCCA |
| PTGS2 | AGGGTTGCTGGTGGTAGGAA | CATCTGCCTGCTCTGGTCAA |
| NCOA4 | CTTTCAAGATGTAACCGTTGGGA | GCAGAAAGGCTGCTCAACTC |
| IL33 | AAGAACACAGCAAGCAAAGCC | GCCAGAGCGGAGCTTCATA |
| GSDMD | GCAGAAGGGACGTGGTGTTC | TCATCCCTCCGCCCAGTTTA |
| RIPK1 | GCCCCAGCCTTTAGATGAGG | TGAAGCTCAAGAACGCCCAA |
| BAK1 | GCAGGCTGATCCCGTCC | CTGCGGAAAACCTCCTCTGT |
| BID | GACACTGTGAACCAGGAGTGAG | GGAGGAAGCCAAACACCAGTA |
| BAX | GAGCAGCCCAGAGGCG | TGAGACACTCGCTCAGCTTC |
| MCL1 | TTCCAGTAAGGAGTCGGGGT | TGGCCAAAAGTCGCCCTC |
| DRP-1 | GATGCCATAGTTGAAGTGGTGAC | CCACAAGCATCAGCAAAGTCTGG |
| FIS1 | CAAGGAACTGGAGCGGCTCATT | GGACACAGCAAGTCCGATGAGT |
| MFF | CAAGGTTCCAGGCACCGATTTC | GCGACAAAATGCCACGAGCAGA |
| MFN1 | GGTGAATGAGCGGCTTTCCAAG | TCCTCCACCAAGAAATGCAGGC |
| MFN2 | ATTGCAGAGGCGGTTCGACTCA | TTCAGTCGGTCTTGCCGCTCTT |
| OPA1 | GTGGTTGGAGATCAGAGTGCTG | GAGGACCTTCACTCAGAGTCAC |

**Supplementary Fig. 1 MMA elevated in conditions of ischemia**-**reperfusion**

**
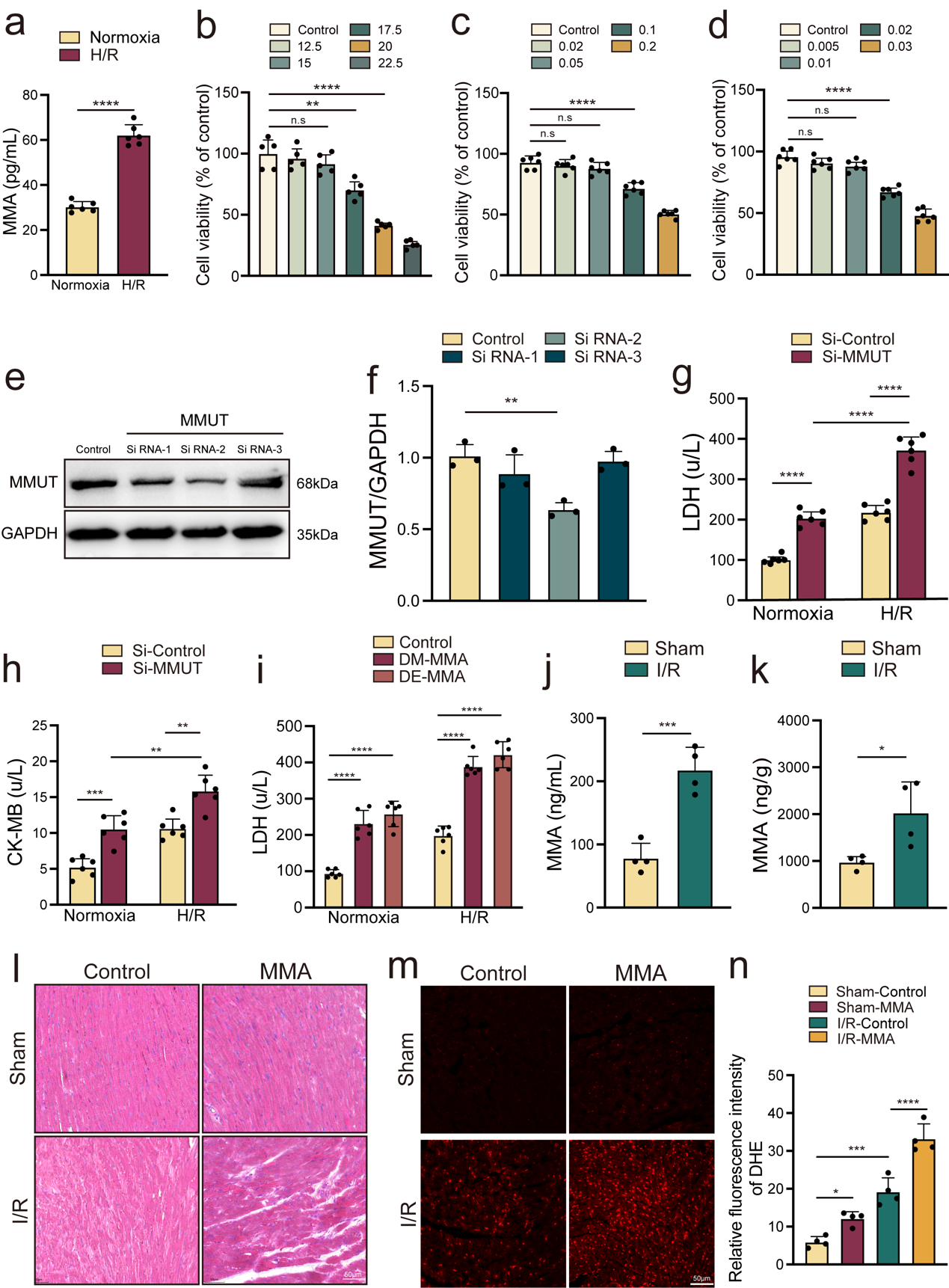
**

**Supplementary Fig. 1 MMA elevated in conditions of** **ischemia-reperfusion.** In vitro, AC16 cells were treated with MMA (17.5mM) for 3h and then used for further analyses. In vivo, mice were assigned to the sham operation or myocardial ischemia-reperfusion (I/R) injury group. MMA (400mg/kg/d) was administered seven days before sham and I/R surgery. **a** MMA level of AC16 cells significantly increased in the H/R context compared with the Normoxia group (n = 4/group). **b-d** AC16 cells were treated with various concentrations of MMA (12.5, 15, 17.5, 20, 22.5mM), DM-MMA (Dimethyl methylmalonate) (0.02, 0.05, 0.1, 0.2μM) or DE-MMA (Diethyl Methylmalonate) (0.005, 0.01, 0.02, 0.03μM) for 3h and then the cell viability was detected by MTT assay (n = 5-6/group). **e-f** AC16 cells were transfected with negative control siRNA or three MMUT siRNAs. WB analyzed the efficiency of silence (n = 3/group). **g-h** AC16 cells were treated with/without MMA in Normoxia or H/R condition. Cellular injury was indicated as CK-MB and LDH assay (n =6/group). **i** AC16 cells were treated with PBS, DE-MMA or DM-MMA in Normoxia or H/R condition. Cellular injury was indicated as lactate dehydrogenase (LDH) assay (n =6/group). **j-k** MMA level of mice serum and mice myocardial tissue significantly increased in the I/R model compared with the sham group (n = 4/group). **l** Representative images of heart sections with HE staining (n =4/group) (Scale bar: 50 μm). **m-n** Representative photomicrographs and averaged data of DHE in mice heart tissue (n = 4/group) (Scale bar: 50 μm). Data are expressed as the mean ± standard deviation. **P* < 0.05, ***P* < 0.01, ****P* < 0.001, *****P* < 0.0001 and *n.s*, not significant.

**Supplementary Fig. 2 MMA affected mitochondrial dynamics.**


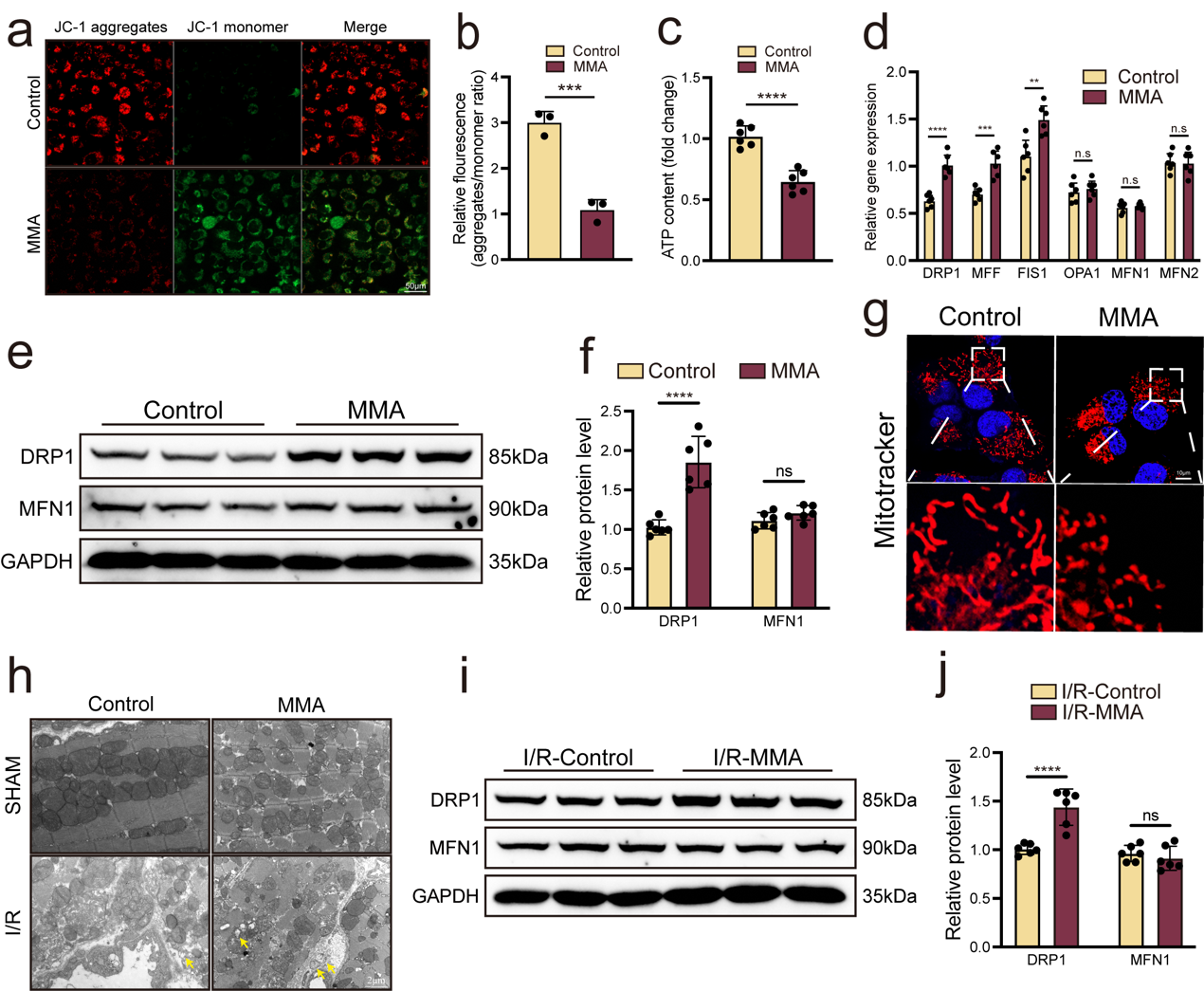


**Supplementary Fig. 2 MMA affected mitochondrial dynamics.** In vitro, AC16 cells were treated with MMA (17.5mM) for 3h and then used for further analyses. In vivo, mice were assigned to the sham operation or myocardial I/R injury group. MMA (400mg/kg/d) was administered seven days before the I/R injury. **a-b** Mitochondrial membrane potential was observed by JC-1 staining (n = 6/group) (Scale bar: 50 μm). **c** Cellular energy production was indicated as ATP assay (n = 6/group). **d** Real-time quantitative PCR (RT-qPCR) analyzed the gene level of DRP1, MFF, FIS1, OPA1, MFN1 and MFN2 (n = 6/group). **e-f** Western blot analysed DRP1 and MFN1 in AC16 cells (n = 6/group). **g** Representative images of mitochondria staining. (Scale bars: 10 μm). **h** TEM analysis of mitochondria in mice heart tissue. Yellow arrows indicate mitochondria (Scale bar: 2 μm). **i-j** Western blot analysed DRP1 and MFN1 of mice heart tissue in I/R model (n = 6/group). Data are expressed as the mean ± standard deviation. **P* < 0.05, ***P* < 0.01, ****P* < 0.001, *****P* < 0.0001 and *n.s*, not significant.

**Supplementary Fig. 3** **MMA and H2O2 caused cell damage**


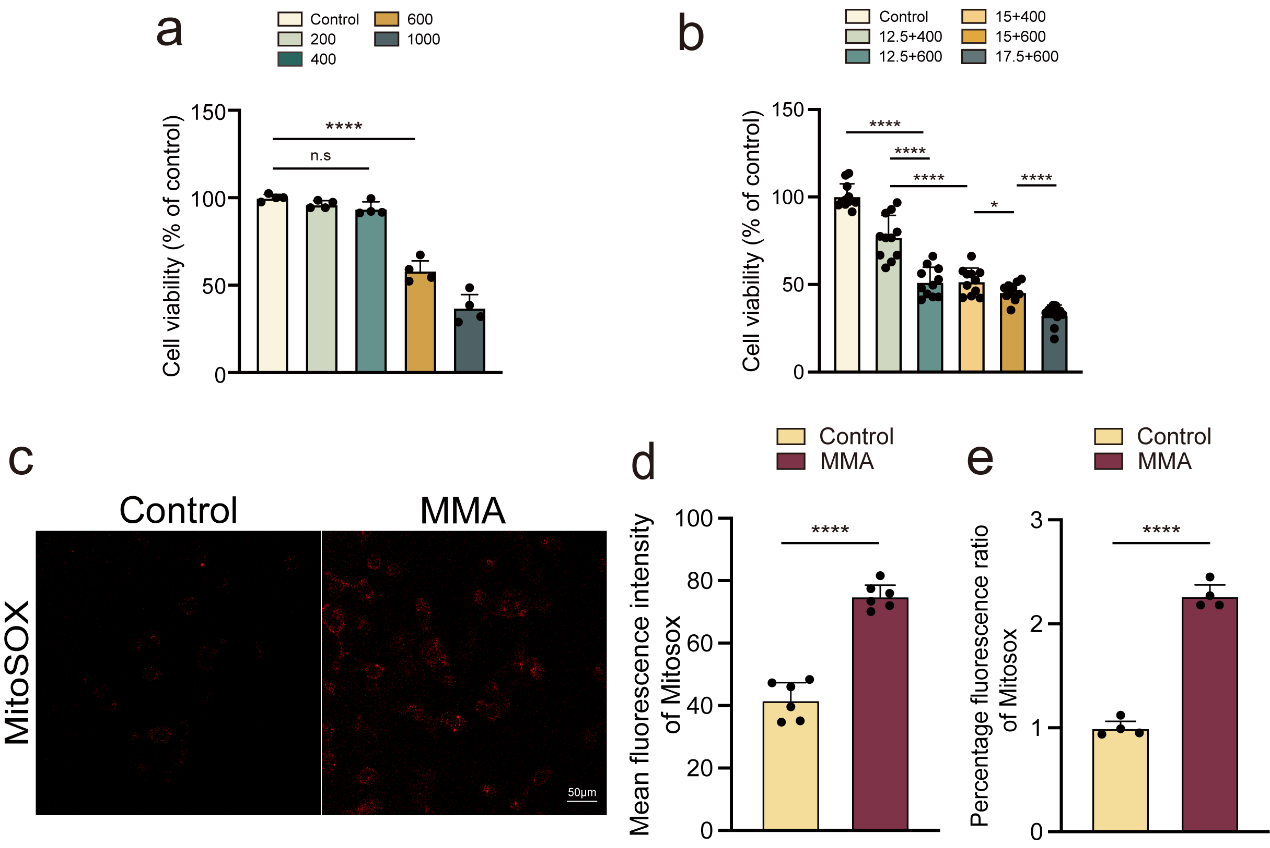


**Supplementary Fig. 3** **MMA and H2O2 caused cell damage. a** AC16 cells were treated with various concentrations of H_2_O_2_ (200, 400, 600, 1000μM) for 6h and then the cell viability was detected by MTT assay (n = 4-5/group). **b** AC16 cells were treated with various concentrations of MMA and H_2_O_2_ simultaneously (12.5+400, 12.5+600, 15+400, 15+600, 17.5+600mM+μM) for 3h. MTT assay was used to detect the cell viability (n = 11/group). **c-d** Mitochondrial oxidative stress was observed by MitoSOX probe (n =3/group) (Scale bar: 50 μm). **e** MMA increased the percentage of MitoSOX-positive cells detected by flow cytometry (n = 4/group). Data are expressed as the mean ± standard deviation. **P* < 0.05, ***P* < 0.01, ****P* < 0.001, *****P* < 0.0001 and *n.s*, not significant.

**Supplementary Fig. 4 NAC, Fer-1 and RSL3 did not affect myocardial function.**

**
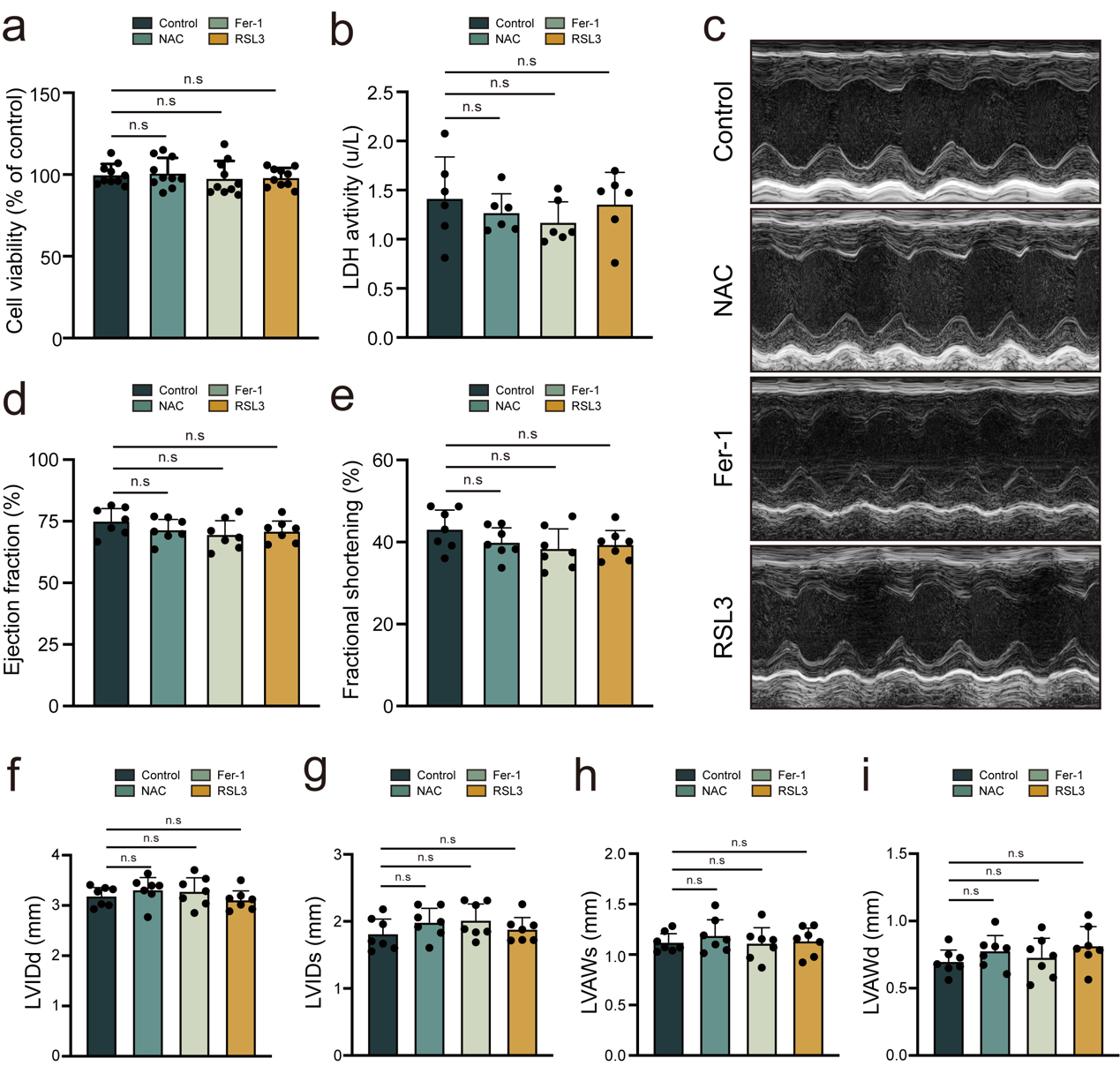
**

**Supplementary Fig. 4 NAC, Fer-1 and RSL3 did not affect myocardial function.** In vitro, AC16 cells were treated with NAC (5mM) for 2h, Fer-1 (2μM) or RSL3 (3μM) for 3h. In vivo, NAC (300mg/kg), Fer-1 (10mg/kg​), and RSL3 (10mg/kg​) were administered on day 2, 4, and 6 of the administration process. **a** Cell viability was detected by MTT assay after after beinig administered with DMSO (dimethyl sulfoxide) as a control, NAC, Fer-1 or RSL3 (n =10/group). **b** Serum levels of myocardial cell death marker LDH in mice treated with NAC, Fer-1, RSL3 and vehicle control (n = 6/group). **c-i** The heart function was represented as EF, FS, left ventricular end-diastolic diameter (LVIDd), left ventricular end-systolic diameter (LVIDs), left ventricular anterior wall; systolic (LVAWs) and left ventricular anterior wall; diastolic (LVAWd) in mice treated with NAC, Fer-1, RSL3 and vehicle control (n = 7/group). Data are expressed as the mean ± standard deviation. **P* < 0.05, ***P* < 0.01, ****P* < 0.001, *****P* < 0.0001 and *n.s*, not significant.

**Supplementary Fig. 5 MMA treatment triggered programmed cell death.**


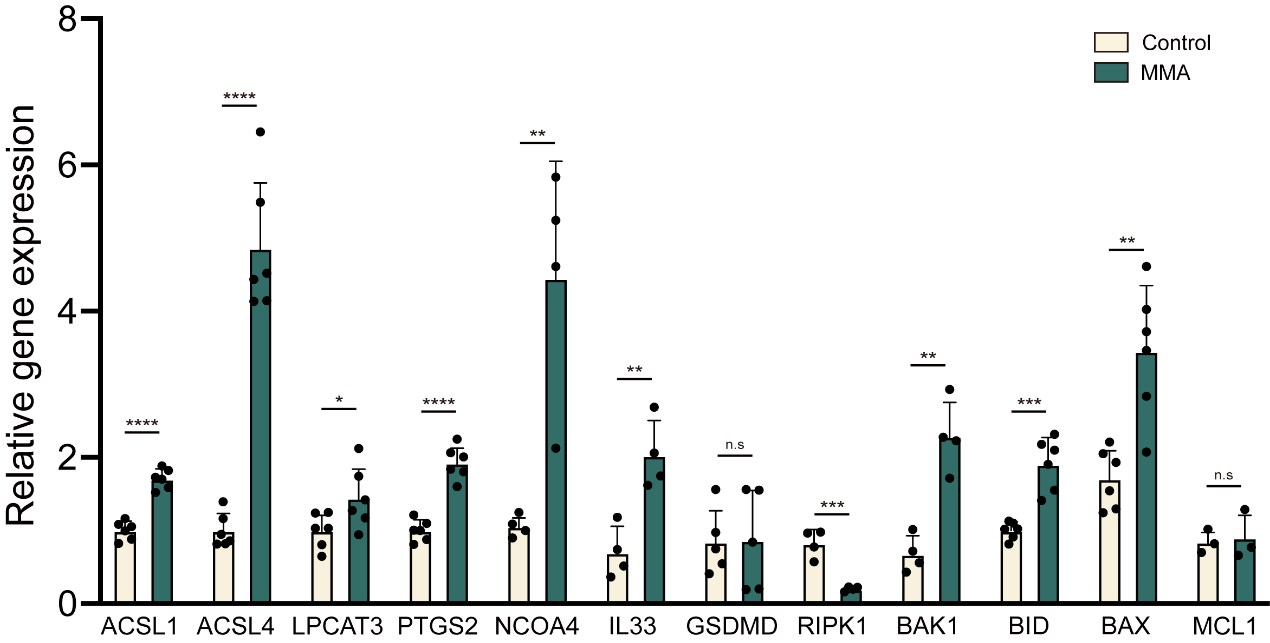


**Supplementary Fig. 5 MMA treatment triggered programmed cell death.** In vitro, AC16 cells were treated with MMA (17.5mM) for 3h and then used for further analyses. RT-qPCR analyzed the gene level of ACSL1, ACSL4, LPCAT3, PTGS2, NCOA4, IL33, GSDMD, RIPK1, BAK1, BID, BAX and MCL1 (n = 3-6/group). Data are expressed as the mean ± standard deviation. **P* < 0.05, ***P* < 0.01, ****P* < 0.001, *****P* < 0.0001 and *n.s*, not significant.

**Supplementary Fig. 6 MitoTEMPO reversed the lipid peroxidation and cell damage caused by MMA**

**
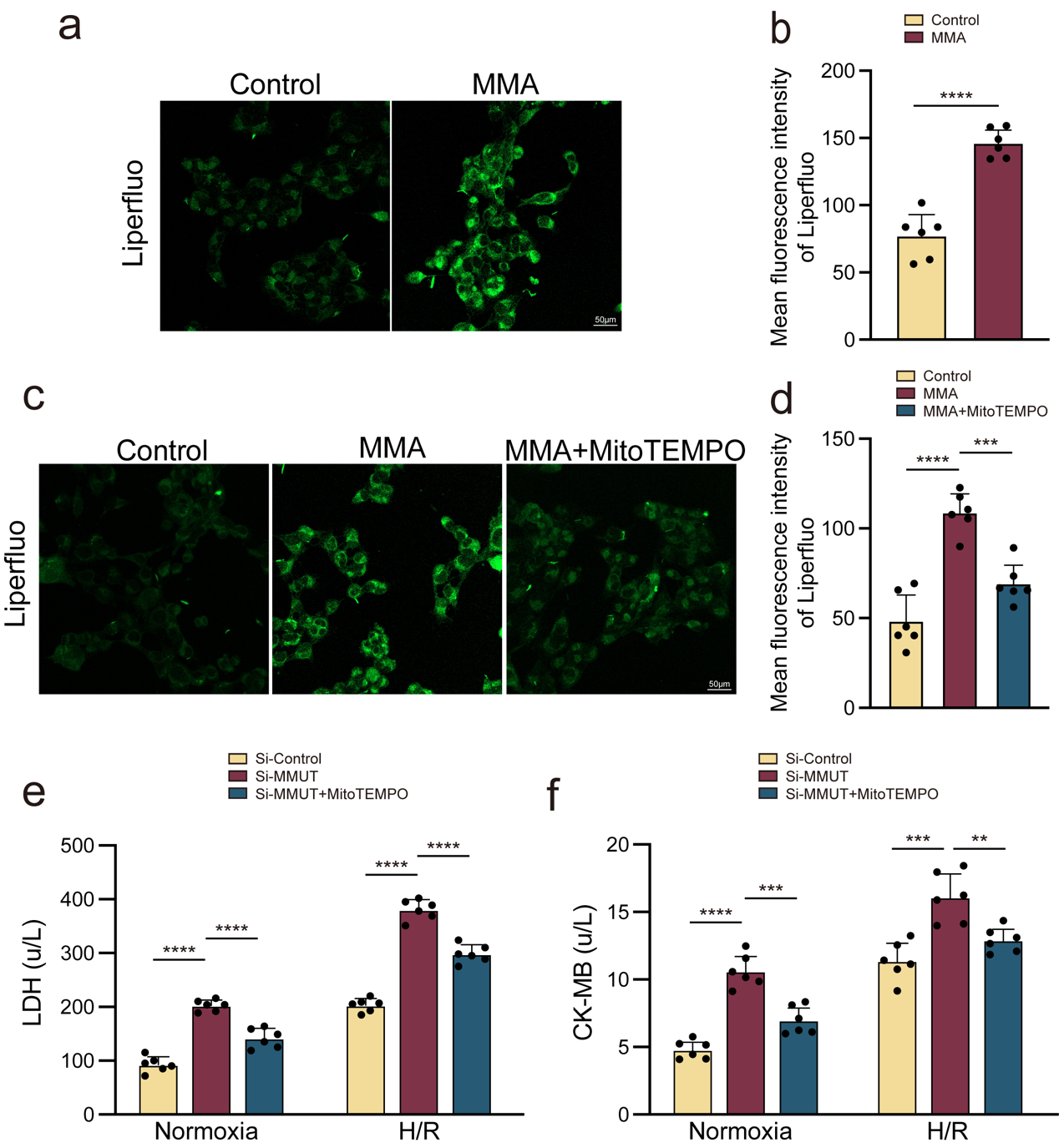
**

**Supplementary Fig. 6 MitoTEMPO reversed the lipid peroxidation and cellular damage caused by MMA**. In vitro, AC16 cells were treated with MMA (17.5mM) alone for 3h or with MitoTEMPO (10μM) for 2h followed by MMA (17.5mM) for 3h. **a-b** Lipid peroxidation was observed by liperfluo probe (n = 6/group) (Scale bar: 50 μm). **c-d** MitoTEMPO decreased lipid peroxidation detected by confocal microscopy (n = 6/group) (Scale bar: 50 μm). **e-f** Cellular injury was inhibited by MitoTEMPO indicated by CK-MB and LDH assay (n = 6/group). Data are expressed as the mean ± standard deviation. **P* < 0.05, ***P* < 0.01, ****P* < 0.001, *****P* < 0.0001 and *n.s*, not significant.

**Supplementary Fig. 7 NRF2 expression at different time points and KEAP1 silence efficiency**

**
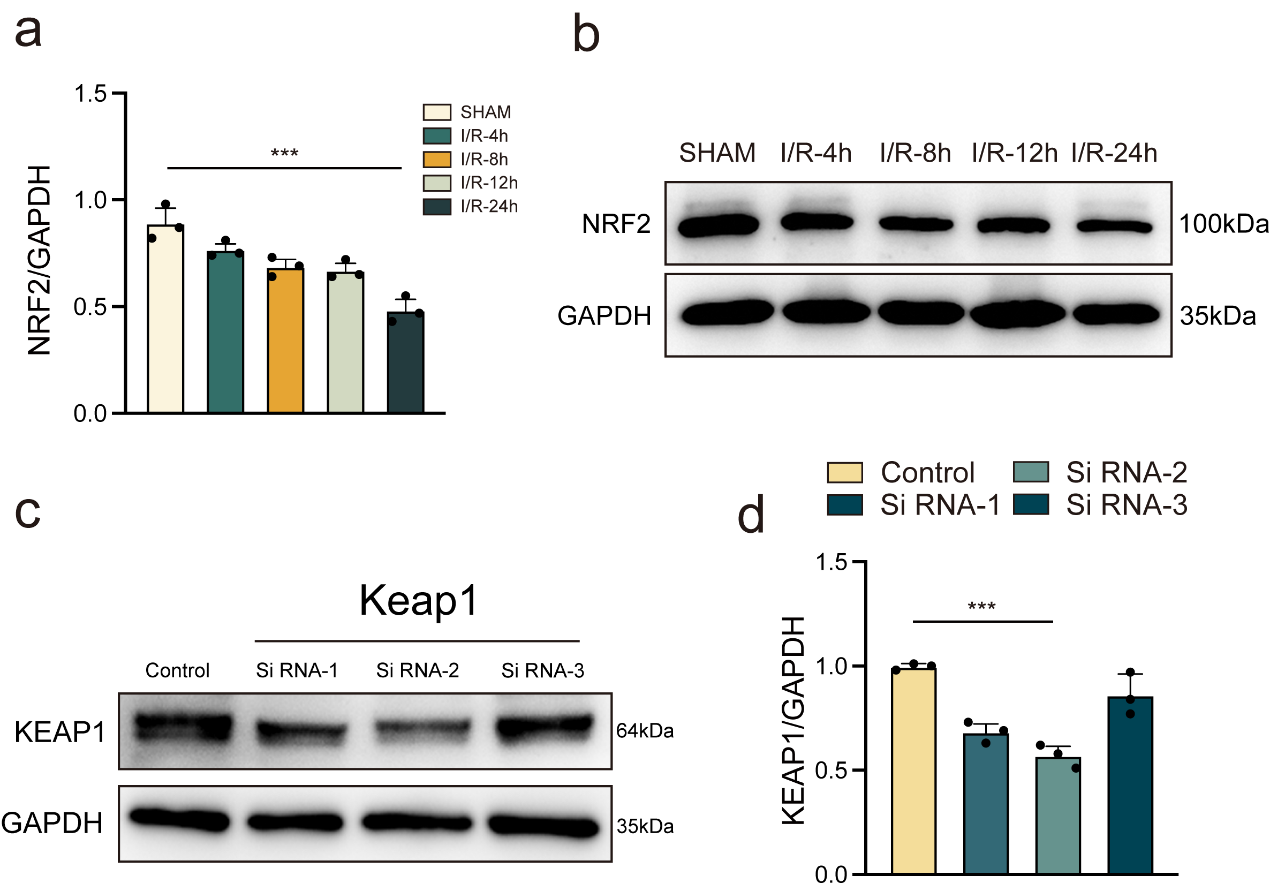
**

**Supplementary Fig. 7 NRF2 expression at different time points and KEAP1 silence efficiency. a-b** Mice were assigned to the sham operation or myocardial I/R injury group. Mice in the I/R group were sacrificed at 4, 8, 12, and 24 hours after reperfusion. Then, western blot analysed NRF2 expression in mice heart tissue (n = 3/group). **c** AC16 were transfected with negative control siRNA or three KEAP1 siRNAs. **d** Western blot analyzed the efficiency of silence (n = 3/group). Data are expressed as the mean ± standard deviation. **P* < 0.05, ***P* < 0.01, ****P* < 0.001, *****P* < 0.0001 and *n.s*, not significant.
